# Acceptor‐Bridged Engineering Enables Highly Efficient Solution‐Processed Pure‐Green MR‐TADF OLEDs With External Quantum Efficiency of 29% and Small Roll‐Off

**DOI:** 10.1002/advs.202518308

**Published:** 2025-11-11

**Authors:** Xuming Zhuang, Qing Zhang, Jinbei Wei, Zhiqiang Li, Baoyan Liang, Chenguang Wang, Hai Bi, Yue Wang, Geyu Lu

**Affiliations:** ^1^ Jihua Laboratory 28 Huandao South Road Foshan Guangdong 528200 China; ^2^ State Key Laboratory of Integrated Optoelectronics, JLU Region, Key Laboratory of Advanced Gas Sensors of Jilin Province, College of Electronic Science and Engineering Jilin University Changchun 130012 China; ^3^ State Key Laboratory of Supramolecular Structure and Materials, College of Chemistry Jilin University Changchun 130012 China

**Keywords:** multiple resonance, narrowband emission, organic light‐emitting diodes, solution‐processed, thermally activated delayed fluorescence

## Abstract

The pursuit of next‐generation ultrahigh‐definition displays has intensified the demand for narrowband emitters with exceptional color purity and high efficiency. Despite the demonstrated superiority of multi‐resonance thermally activated delayed fluorescence (MR‐TADF) emitters in vacuum‐deposited OLEDs, their solution‐processing implementation faces significant challenges stemming from inherent molecular rigidity, which compromises solubility, film morphology, and inefficient exciton utilization. Herein, two novel solution‐processable, pure‐green MR‐TADF emitters, DBN‐Pym and DBN‐PhPym, are reported via an acceptor‐bridged engineering strategy that integrates MR cores with electron‐withdrawing pyrimidine bridges. Such a design simultaneously enhances the solubility through controlled molecular torsion and precisely modulates the electron distribution via synergistic long‐ and short‐range charge transfer. Consequently, the resulting emitters exhibit narrowband green emission (514/513 nm, full width at half maximum, FWHM: 24–30 nm) with remarkably high photoluminescence quantum yields (PLQYs) of 86% and 99%, respectively. Solution‐processed OLEDs exhibit peak emissions at 526/521 nm with Commission Internationale de L'Eclairage (CIE) y coordinates exceeding 0.69. Notably, the DBN‐PhPym achieves a record external quantum efficiency (EQE) of 29.0% (maintaining 28.3% at 1000 cd m^−2^), with an FWHM of 34 nm, representing state‐of‐the‐art performance for solution‐processed MR‐TADF devices. This work establishes an effective molecular design strategy for developing efficient narrowband emitters, paving the way for solution‐processable ultrahigh‐definition displays.

## Graphical abstract

Solution‐processible pure green multi‐resonance thermally activated delayed fluorescence (MR‐TADF) emitters are developed through a strategic acceptor‐bridging engineering approach that integrates MR‐building blocks with pyrimidine‐derived frameworks. The resulting solution‐processed OLEDs achieve a maximum external quantum efficiency (EQE) of 29.0%, while maintaining 28.3% at 1000 cd m^−2^, along with Commission Internationale de I’Éclairage (CIE) coordinates of (0.213, 0.693).

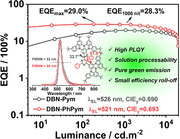

## 1 Introduction

The advancement of organic electroluminescent technology has redefined performance benchmarks in the visual display field, achieving widespread adoption in premium consumer electronics ranging from smartphones and televisions to wearable devices.^[^ ^1^, ^2^, ^3^, ^4^ ^]^ The escalating demand for superior screen performance, such as ultra‐high‐definition display technology, coupled with the accelerated development of near‐eye display technologies including augmented reality (AR) and virtual reality (VR) systems, has created an urgent need for novel emitters with narrow spectral widths.^[^ ^5^, ^6^, ^7^, ^8^, ^9^ ^]^ These advanced materials must simultaneously achieve high color gamut and exceptional luminous efficiency. Multi‐resonance thermally activated delayed fluorescence (MR‐TADF) emitters, pioneered by Hatakeyama's foundational work, have emerged as a leading candidate class due to their exceptional narrowband emission and high efficiency.^[^ ^10^ ^]^ These materials incorporate orthogonally arranged boron (B) centers and nitrogen (N)/oxygen group atoms (O, S, Se) within rigid polycyclic frameworks, the opposite electronic properties of boron and nitrogen/oxygen group atoms induce the highest occupied molecular orbital (HOMO) and lowest unoccupied molecular orbital (LUMO) to be naturally separated in space and alternately populated on atoms.^[^ ^11^, ^12^, ^13^, ^14^ ^]^ This unique molecular architecture effectively suppresses vibrational and rotational perturbations in covalent bonds, thereby minimizing disruptions to excited‐state configurations, which enable precise narrowband emission profiles while maintaining high photoluminescence quantum yields (PLQYs), positioning MR‐TADF materials as a focal point of interdisciplinary research across industrial and academic sectors.

Solution‐processed organic light‐emitting diodes (OLEDs) demonstrate distinct technological advantages, including enhanced material utilization efficiency, scalability for large‐area fabrication, and compatibility with flexible substrate integration, positioning them as competitive alternatives to conventional vacuum‐deposited counterparts.^[^ ^15^, ^16^, ^17^ ^]^ While MR‐TADF materials have achieved remarkable performance in vacuum‐deposited electroluminescent (EL) devices, characterized by narrow full width at half maximum (FWHM <35 nm) and high external quantum efficiencies (EQE >30%),^[^ ^18^, ^19^ ^]^ their adaptation to solution‐processable architectures remains impeded by rigid planar molecular configurations that compromise solubility, film morphology, and inefficient exciton utilization. Current strategies to enhance solution processability of MR‐TADF materials predominantly focus on two approaches: 1) peripheral auxiliary units induce conformational flexibility but often trigger strong intramolecular charge transfer (ICT) effects, leading to spectral broadening with unsatisfied color purity;^[^ ^20^, ^21^, ^22^ ^]^ 2) covalent integration of multiple MR‐TADF cores through bridge engineering enables controlled molecular twisting, as demonstrated by dual‐BN‐unit emitter bridged by substituted carbazole linkers^[^ ^23^ ^]^ and triple‐BN‐unit design incorporating three MR units on phenyl scaffolds.^[^ ^24^ ^]^ Although these methodologies enhance solution processability and achieve notable electroluminescent (EL) performance, persistent challenges in spectral control with the desired emission color impede their practical implementation, particularly in high‐resolution panel manufacturing. Consequently, the simultaneous optimization of narrowband emission, high efficiency, and solution processability remains a critical challenge for developing high‐performance green and red MR‐TADF materials.

**Scheme 1:**
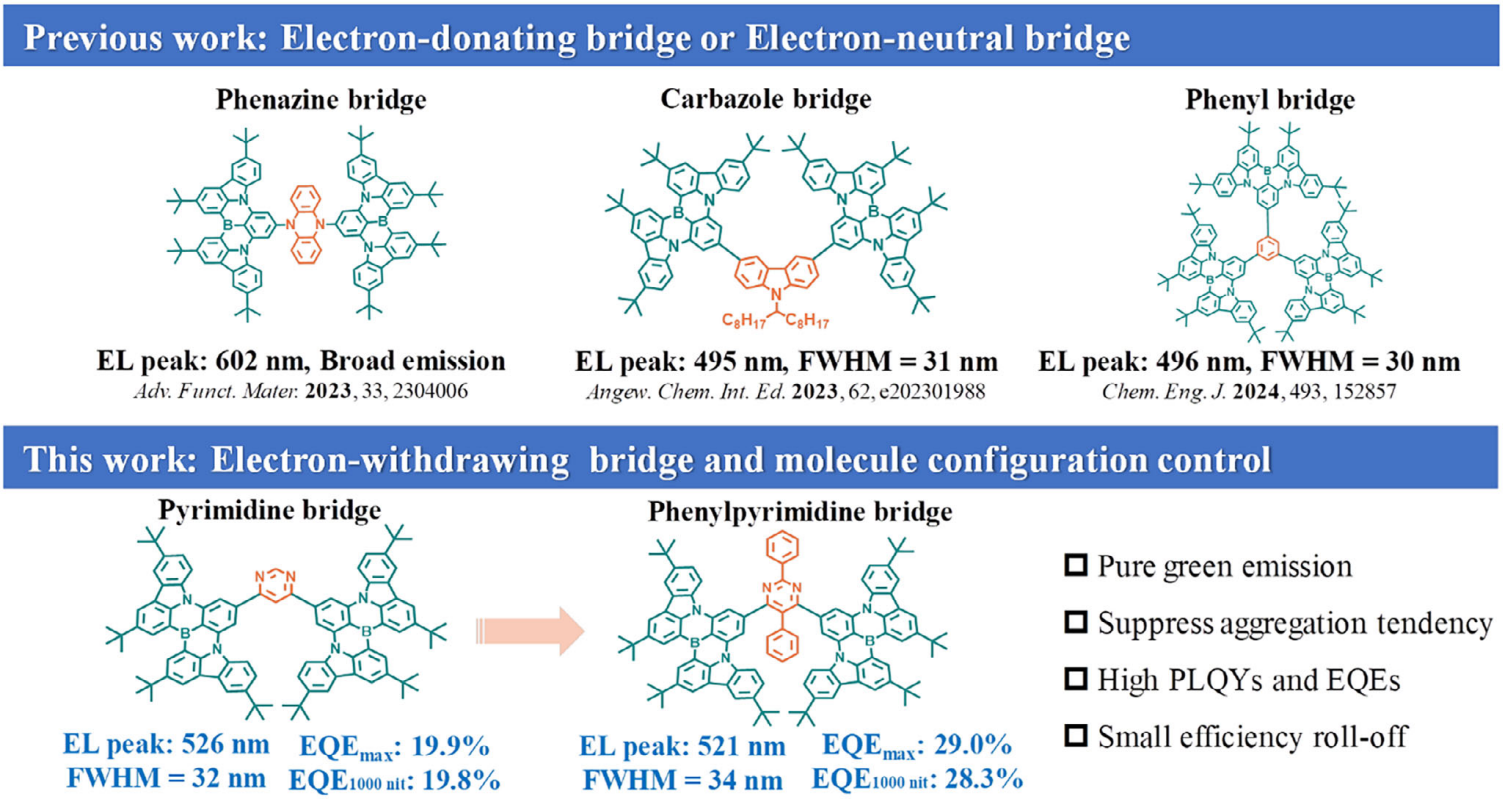
Molecular design strategy of DBN‐Pym and DBN‐PhPym.

In this work, we developed two solution‐processable MR‐TADF emitters, namely DBN‐Pym and DBN‐PhPym, through acceptor‐bridged engineering that strategically integrates BNCz‐based MR‐building blocks with pyrimidine‐derived bridges, achieving synergistic enhancement in multiple dimensions. (**Scheme** **1**). The bluish‐green BNCz core was selected for its superior EL performance and inherent MR effect characteristics.^[^ ^25^, ^26^, ^27^ ^]^ Building on this, the pyrimidine ring functions as both a structural linker, introducing controlled torsion between BNCz units to enhance solution processability, and an electron distribution modulator, facilitating long‐range charge transfer (LRCT) transitions.^[^ ^28^, ^29^, ^30^ ^]^ The LRCT synergizes with BNCz‐core dominant short‐range charge transfer (SRCT) processes to achieve pure green emission while preserving narrowband emission. Through systematic molecular optimization, compounds DBN‐Pym and DBN‐PhPym exhibit green emission in toluene with peak maxima at 514 and 513 nm, FWHM values of 24 and 30 nm, and PLQYs of 86% and 99%, respectively. As anticipated, both DBN‐Pym and DBN‐PhPym exhibit improved solubility and film‐forming ability. Optimized solution‐processed sensitized OLEDs incorporating these emitters manifest pure green electroluminescence with peak wavelengths at 526 and 521 nm, accompanied by Commission Internationale de L'Eclairage (CIE) coordinates of (0.256, 0.690) and (0.213, 0.693), and maximum external quantum efficiencies (EQEs) of 19.9% and 29.0%. Notably, these devices maintain EQEs of 19.8% and 28.3% at a practical brightness of 1000 cd m^−2^. Collectively, the demonstrated device performance represents a state‐of‐the‐art achievement for solution‐processed OLED technology, establishing a robust molecular design paradigm for high‐efficiency solution‐processable MR‐TADF emitters.

## 2 Results and Discussion

### 2.1 Synthesis, Thermostability, and Electrochemistry Properties

The molecular architecture and design rationale are illustrated in Scheme 1. The target green MR‐TADF emitter was constructed by integrating two established BNCz moieties featuring typical SRCT characteristics through a phenyl‐pyrimidine bridging acceptor. Steric hindrance between BNCz and the phenyl groups on the bridge induces pronounced molecular torsion, thereby significantly improving solution processability and mitigating aggregation. Simultaneously, the electron‐withdrawing pyrimidine groups facilitate the LRCT processes, which optimize the energy levels and lead to a pure green emission. As shown in Scheme S1 (Supporting Information), DBN‐Pym and DBN‐PhPym are synthesized through a Suzuki‐Miyaura cross‐coupling reaction with key intermediate BNCz‐Bpin.^[^ ^31^ ^]^ Both emitters were characterized by ^1^H nuclear magnetic resonance spectroscopy (NMR), ^13^C NMR, and Matrix‐Assisted Laser Desorption/Ionization Time‐of‐Flight (MALDI‐TOF) mass spectrometry as depicted in Figures S1–S6 (Supporting Information). Additionally, both compounds exhibit good solubility in common organic solvents (e.g., chlorobenzene, o‐xylene, toluene, methyl benzoate), which facilitates their use in solution‐processed OLED fabrication.

Thermogravimetric analysis (TGA) showed high thermal decomposition temperatures (*T*_d_s, corresponding to 5% weight loss) of 507.5 °C and 524.1 °C for the two compounds, respectively, confirming their exceptional thermal stability. Differential scanning calorimetry (DSC) measurements revealed glass transition temperatures (*T_g_* s) above 220 °C, endowing the material with sufficient thermal stability to withstand essential solution‐processing steps like thermal annealing (Figure S9, Supporting Information). The HOMO energy levels of DBN‐Pym and DBN‐PhPym were assigned as −5.37 and −5.36 eV via cyclic voltammetry (CV) measurement (Figure S10, Supporting Information), while the LUMO energy levels were derived as −2.99 and −2.94 eV by combining the HOMO values with the optical bandgap. Both compounds exhibit relatively small HOMO‐LUMO energy gaps (*E*_gap_s), with a value of 2.38 eV for DBN‐Pym and 2.42 eV for DBN‐PhPym. These energy levels demonstrate compatibility with conventional organic host matrices.

### 2.2 Theoretical Calculation and Configuration Analysis

**Figure 1:**
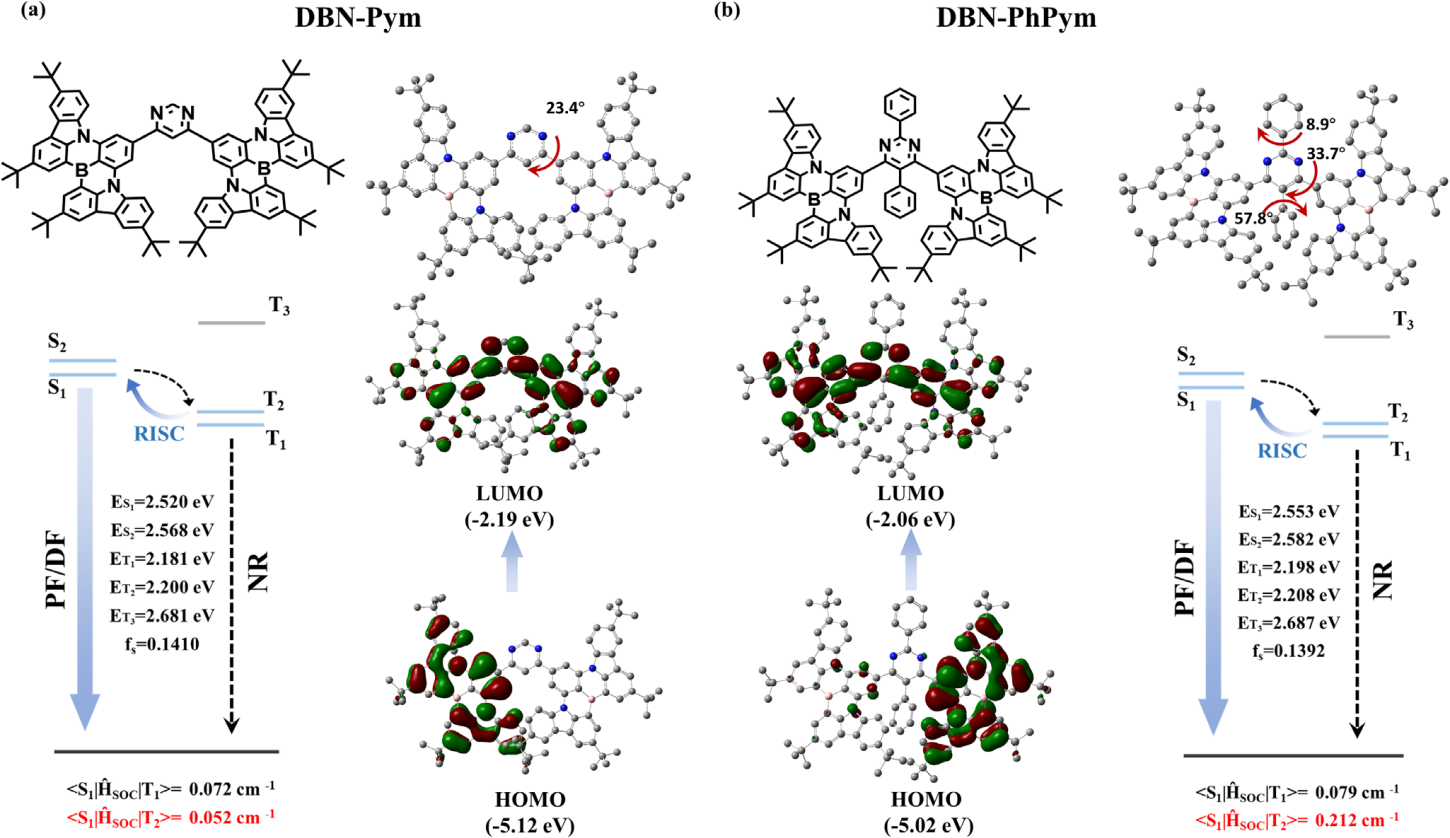
Optimized ground state (S_0_) structure and HOMO/LUMO distributions of a) DBN‐Pym and b) DBN‐PhPym.

To elucidate the frontier molecular orbital (FMO) distributions, geometric configurations, and electronic properties of DBN‐Pym and DBN‐PhPym, density functional theory (DFT) and time‐dependent DFT (TD‐DFT) calculations were performed at the B3LYP/6‐31G (d, p) level. In the DBN‐Pym molecule, the dihedral angle between the MR‐emitting unit and the central pyrimidine acceptor is 23.4° (**Figure** **1**; Figure S11, Supporting Information), which introduces moderate steric hindrance and partially reduces molecular planarity. Upon introduction of two phenyl rings to the central pyrimidine, the resulting DBN‐PhPym exhibits significantly larger dihedral angles: 33.7° between the MR‐emitting unit and the pyrimidine ring, and 57.8° between the phenyl ring and the pyrimidine ring. This more twisted molecular conformation is expected to promote solubility and suppress aggregation‐caused quenching (ACQ) by preventing tight molecular packing. Consequently, DBN‐PhPym demonstrates a higher solid‐state luminescence efficiency than DBN‐Pym. In both compounds, the HOMOs are mainly localized on the donor nitrogen atom and carbon atoms at ortho/para‐positions, while the LUMOs are predominantly distributed over the boron atoms and adjacent ortho‐/para‐carbons in the BNCz moiety, facilitating SRCT in typical MR‐TADF emitters. Accompanying this electronic arrangement is a partial delocalization of the LUMO onto the pyrimidine acceptor unit, introducing a contribution from LRCT (Figure 1). This hybridized electronic feature supports not only narrowband emission but also a tunable bathochromic shift in fluorescence.

Furthermore, the natural transition orbitals (NTOs) of the excited singlet (S_n_, n = 1,2) and triplet (T_n_, n = 1,2) states of DBN‐Pym and DBN‐PhPym were also calculated using the Multiwfn code (Figure S12, Supporting Information). The calculated hole and particle distributions follow the characteristic MR pattern, consistent with previously reported MR‐type emitters. Specifically, the hole density is predominantly localized on the BNCz framework, whereas the particle density extends onto the pyrimidine ring. The NTO analysis reveals a delocalization pattern that manifests the hybrid nature of the excited states, which intricately combine LRCT and MR features. Notably, we observed that DBN‐Pym and DBN‐PhPym exhibit higher excited states (S_2_/T_2_) with energies of 2.568/2.200 eV and 2.582/2.208 eV, respectively, which are energetically close to their corresponding S_1_/T_1_ states (2.520/2.181 eV for DBN‐Pym and 2.553/2.198 eV for DBN‐PhPym). This near‐degeneracy between excited states enhances state mixing, facilitating the rapid reverse intersystem crossing (RISC) process. This phenomenon arises from the hybrid nature of these excited states, which results from a combination of both SRCT within the individual BNCz unit and LRCT between the BNCz donor and the pyrimidine acceptor. The calculated spin‐orbit coupling (SOC) matrix elements reveal that <S_1_|*Ĥ*_SOC_|T_1_> values are comparable for both compounds (0.072 cm^−1^ for DBN‐Pym versus 0.079 cm^−1^ for DBN‐PhPym). However, a notable difference was observed in the <S_1_|*Ĥ*_SOC_|T_2_> values. The coupling constant of DBN‐PhPym (0.212 cm^−1^) was significantly larger than that of DBN‐Pym (0.052 cm^−1^), leading to a more efficient RISC process.

### 2.3 Photophysical Properties

**Figure 2:**
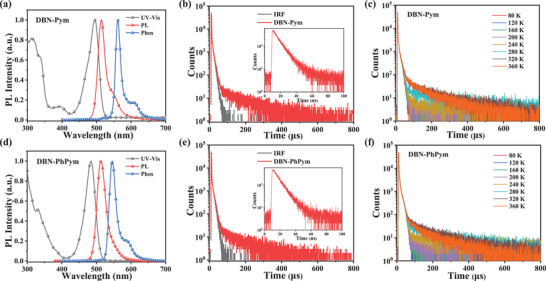
a,d) Ultraviolet‐visible (UV–vis) absorption, fluorescence (298 K), and phosphorescence (77 K) spectra of DBN‐Pym and DBN‐PhPym; b,e) Transient photoluminescence decay curves of DBN‐Pym and DBN‐PhPym in mCP host matrix at a doping concentration of 2 wt%; c,f) Temperature‐dependent time‐resolved photoluminescence spectra of DBN‐Pym and DBN‐PhPym in mCP host matrix at a doping concentration of 2 wt% under different temperatures from 80 to 360 K.

The photophysical properties of DBN‐Pym and DBN‐PhPym were characterized by ultraviolet‐visible (UV–vis) absorption and photoluminescence (PL) spectroscopy in dilute toluene solution at 298 K, complemented by low‐temperature (77 K) phosphorescence measurements (**Figure** **2**a,d and **Table** **1**). Both compounds exhibited intense, narrow absorption bands at 495 nm (DBN‐Pym) and 484 nm (DBN‐PhPym), respectively, attributed to SRCT transitions within the BNCz MR framework. The corresponding fluorescence emissions exhibited peaks at 514 nm for DBN‐Pym and 513 nm for DBN‐PhPym, with narrow FWHMs of 24 nm and 30 nm, respectively. Compared to the parent BNCz moiety, both DBN‐Pym and DBN‐PhPym exhibit a distinct redshift, which is attributed to the expansion of the LUMO and the emergence of an LRCT character. Notably, DBN‐PhPym demonstrated a broader emission profile than DBN‐Pym, which we ascribe to its enhanced LRCT contribution, which is corroborated by its larger dihedral angles (33.7°/57.8° versus 23.4° for DBN‐Pym). DBN‐Pym and DBN‐PhPym exhibited high PLQYs of 86% and 99%, respectively, in N_2_‐bubbling toluene. The lower PLQY of DBN‐Pym likely stems from its reduced steric hindrance, which promotes non‐radiative decay through enhanced molecular rotation and vibrational relaxation. The S_1_/T_1_ energy levels (*E*_S1_/*E*_T1_) determined from the fluorescence and phosphorescence spectra in frozen toluene were measured at 2.41/2.20 eV for DBN‐Pym and 2.42/2.27 eV for DBN‐PhPym. The corresponding singlet‐triplet energy gaps (Δ*E*_ST_s) were calculated as 0.21 and 0.15 eV, respectively. These small Δ*E*_ST_ values (<0.25 eV) are indicative of efficient TADF via RISC from T_1_ to S_1_.

**Table 1:** Summary of the photophysical properties of DBN‐Pym and DBN‐PhPym.

Emitter	*λ*_abs_ [nm]^a)^	*λ*_em_ [nm]^b)^	FWHM [nm]^c)^	*E*_S1_ [eV]^d)^	*E*_T1_ [eV]^e)^	Δ*E*_ST_ [eV]^f)^	*E*_g_ [eV]^g)^	τ*_p_* [ns]	τ_d_ [µs]	HOMO [eV]^h)^	LUMO [eV]^i)^	PLQY [%]^j)^
DBN‐Pym	495	514	24	2.41	2.20	0.21	2.38	8.7	53.3	−5.37	−2.99	86
DBN‐PhPym	484	513	30	2.42	2.27	0.15	2.42	8.6	44.0	−5.36	−2.94	99

^a)^ Peak wavelength of the lowest energy absorption band.

^b)^ Peak wavelength of the PL spectrum in toluene (1 × 10^−5^ M, 298 K).

^c)^ Full width at half maximum of the PL spectrum.

^d)^ Singlet energy estimated from the peak of the fluorescence spectrum in toluene (10^−5^ M, 298 K).

^e)^ Triplet energy estimated from the peak of the phosphorescence spectrum in a frozen toluene matrix (10^−5^ M, 77 K).

^f)^ Δ*E*_ST_ = *E*_S1_ − *E*_T1_.

^g)^ Optical band gap estimated from the absorption edge of the UV–vis spectrum.

^h)^ Determined from cyclic voltammetry using the formula: *E*_HOMO_ = − (*E*_ox_ + 4.8) eV,

^i)^ *E*_LUMO_═ *E*_HOMO_+*E*_g_.

^j)^ Absolute photoluminescence quantum yield measured with an integral‐sphere system in N_2_‐bubbling toluene.

**Figure 3:**
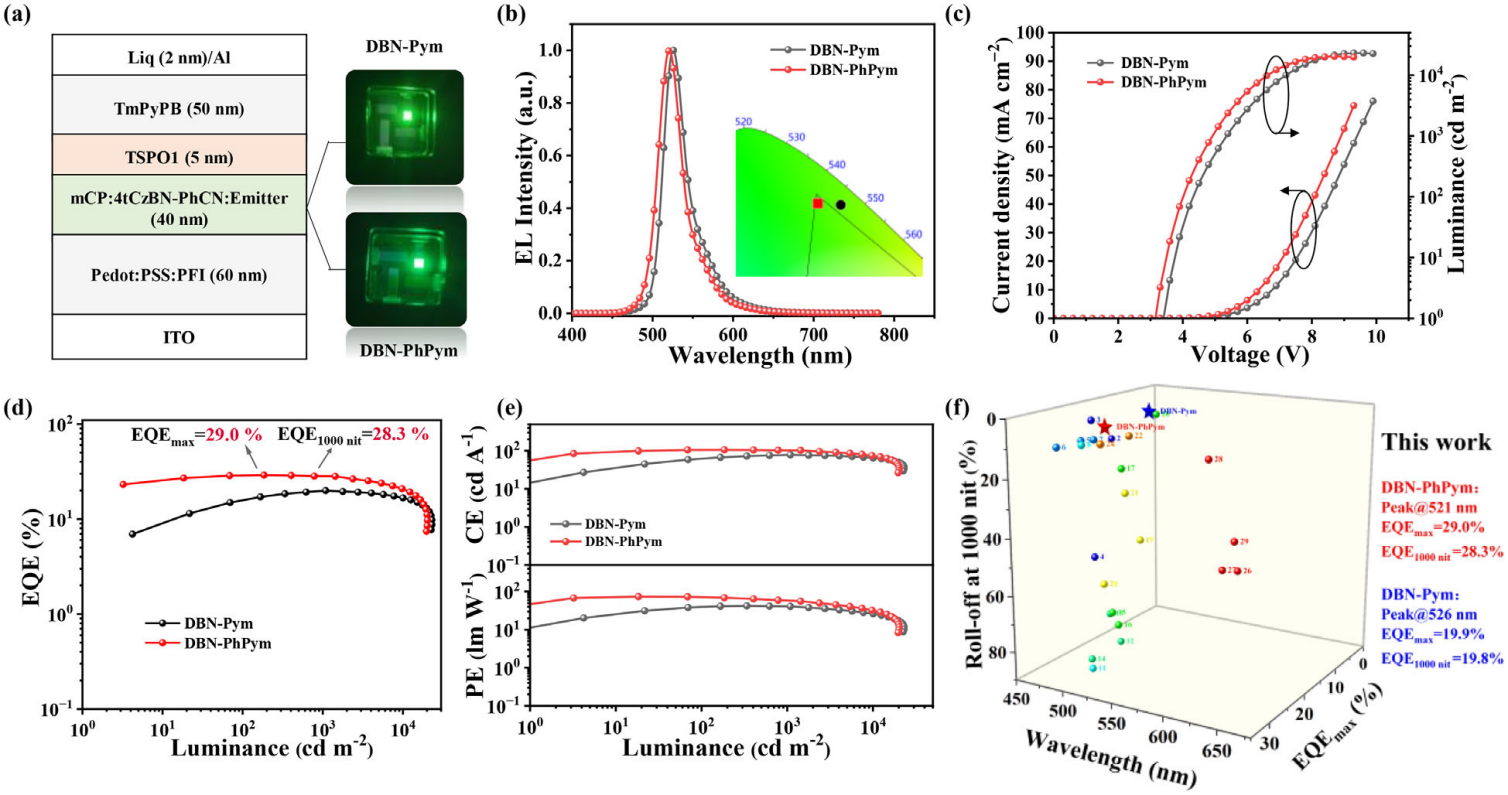
a) The Device configurations and photograph, b) normalized EL spectra, c) Current density–Voltage–Luminescence (*J*–*V*–*L*) curves, d) EQE‐luminance curve, e) Current efficiency–Luminescence (*CE*–*L*) and Power efficiency–Luminescence (*PE*–*L*) curves of the sensitized sOLEDs. f) Device performance comparison graph of the MR‐TADF based sOLEDs.

To elucidate solid‐state emission performance, spin‐coated thin films of DBN‐Pym and DBN‐PhPym (2 wt% doped in 1,3‐bis(*N*‐carbazolyl)benzene (mCP) host) were studied using atomic force microscopy (AFM) and time‐resolved photoluminescence (TRPL) spectroscopy. The excellent film‐forming properties were confirmed by AFM analysis, which revealed highly uniform morphologies with small root‐mean‐square (RMS) roughness values of 0.555 nm (DBN‐Pym) and 0.602 nm (DBN‐PyPym) (Figure S13, Supporting Information). An analogous compound, SBN‐PhPym, containing only one BNCz chromophore, was also synthesized to evaluate the effect of the twisted molecular structure on film‐forming capability. Its molecular structure was confirmed by ^1^H NMR and MALDI‐TOF mass spectrometry (Figures S7 and S8, Supporting Information). SBN‐PhPym exhibited green fluorescence with an emission maximum at 512 nm and a FWHM of 23 nm (Figure S14, Supporting Information). In contrast to DBN‐Pym and DBN‐PhPym, however, SBN‐PhPym demonstrated a planar structure with significantly poorer solubility (Figures S11 and S15, Supporting Information). The roughness values of the films based on DBN‐Pym and DBN‐PhPym were lower than those of films derived from the parent BNCz and SBN‐PhPym. indicating an improvement in film quality attributable to the twisted structure. Precisely controlled covalent assembly of multiple MR‐TADF units thus provides an efficient strategy for constructing solution‐processable emitters that simultaneously achieve narrowband emission and enhanced film‐forming capabilities. Additionally, angle‐resolved and polarization‐resolved PL measurements determined an approximately random molecular orientation for both DBN‐Pym and DBN‐PhPym in the spin‐coated films (Figure S16, Supporting Information). TRPL measurements revealed significant delayed components with short exciton lifetimes in both materials, confirming efficient TADF behavior and rapid reverse intersystem crossing characteristics. The prompt (τ_p_) and delayed (τ_d_) fluorescence lifetimes of two materials are 8.7 ns and 53.3 µs for DBN‐Pym and 8.6 ns and 44.0 µs for DBN‐PyPym, respectively (**Figure** **3**b,e and Table 1). The doped films maintained high PLQY values of 75% for DBN‐Pym and 99% for DBN‐PhPym, demonstrating excellent emission efficiency in the solid state. Temperature‐dependent TRPL analysis further confirmed the TADF characteristics of these emitters. As shown in Figure 2c,f, the delayed fluorescence component exhibited an enhancement with increasing temperature from 80 K to 360 K, which is a distinguishing signature of TADF behavior. To probe the singlet–triplet exciton dynamics of the two TADF emitters in doped films, key rate constants were determined based on lifetime and quantum yield measurements, including those for singlet radiative decay (*k*_s_), nonradiative decay (*k*_nr_), intersystem crossing (*k*_ISC_), and reverse intersystem crossing (*k*_RISC_) (Table S1, Supporting Information). The *k*_RISC_ value of DBN‐PhPym is significantly larger than that of DBN‐Pym, which is consistent with the calculated results. Moreover, to evaluate aggregation behavior, we prepared solution‐processed films of DBN‐Pym and DBN‐PhPym in an mCP host matrix with varying doping concentrations (2, 5, and 10 wt%). As shown in Figure S17 (Supporting Information), as the doping concentration increased from 2 wt% to 10 wt%, DBN‐Pym exhibited a redshift in its emission spectrum from 525 to 530 nm, accompanied by a broadening of the FWHM from 34 to 41 nm. In contrast, DBN‐PhPym showed only a minimal spectral shift (from 523 to 527 nm) and maintained a constant FWHM of 34 nm. The enhanced spectral stability of DBN‐PhPym can be attributed to its increased torsional rigidity, which effectively suppresses intermolecular aggregation and minimizes undesirable spectral changes. The transient photoluminescence decay curves of DBN‐Pym and DBN‐PhPym in an mCP host matrix at different doping concentrations are also shown in Figure S18 (Supporting Information).

### 2.4 Electroluminescence Performance

The exceptional photophysical properties and solution‐processability of DBN‐Pym and DBN‐PhPym prompted a comprehensive investigation of their EL performance. We fabricated OLED devices with solution‐processed emitting layers (EMLs) incorporating DBN‐Pym and DBN‐PhPym dopants within 9‐(3‐(9*H*‐carbazol‐9‐yl)phenyl)‐9*H*‐3,9′‐bicarbazole (mCPBC) host matrix, using the following device configuration: indium tin oxide (ITO)/(PEDOT:PSS):PFI (60 nm)/ mCPBC: emitter x wt% (40 nm)/ DMFBD‐TRZ (5 nm)/Na‐An‐BI:Liq (50 nm)/Liq (2 nm)/Al (80 nm). Here, indium tin oxide (ITO) and aluminum (Al) served as the anode and cathode, respectively, poly(3,4‐ethylenedioxythiophene) polystyrene sulfonate (PEDOT:PSS) mixed with perfluorinated resin (PFI) functioned as the hole injection layer.^[^ ^32^, ^33^ ^]^ mCPBC, DMFBD‐TRZ, and Liq served as host matrix, hole blocking, and electron injection layers. Na‐An‐BI:Liq with a ratio of 5:5 was employed as the electron transport layer. The corresponding device architecture and molecular structures are depicted in Figure S19 (Supporting Information). We systematically evaluated devices with varying doping concentrations (2, 5, and 10 wt%), with all performance metrics, including EL spectra, EQEs – luminance (*L*) characteristics, current density (*J*) ‐ voltage (*V*) – *L* behavior, and current efficiency (CE) and power efficiency (PE) – *L* relationships, which were presented in Figures S21 and S22 and Table S2 (Supporting Information). Both emitters demonstrated pure green EL properties, with DBN‐Pym exhibiting EL peaks of 527–531 nm and emission broadening (FWHM values from 32 nm to 41 nm) as the doping concentrations increased from 2 to 10 wt%. In contrast, DBN‐PhPym maintained exceptional spectral stability, maintaining nearly constant FWHM values (33–34 nm) across all doping concentrations. This consistency with their PL behavior provides direct evidence that the enhanced torsional strain effectively suppresses concentration quenching and spectral broadening. Remarkably, DBN‐PhPym‐based devices achieved CIE_y_ coordinates exceeding 0.700, representing a rare achievement for solution‐processed green OLEDs.^[^ ^34^ ^]^ Despite their excellent optical characteristics, the devices exhibited relatively low EQEs of 10.3% (DBN‐Pym) and 12.1% (DBN‐PhPym), which could be attributed to the prolonged excited‐state lifetime, suboptimal *k*_RISC_, and compromised carrier transport properties within the emitting layers.

To enhance the performance of solution‐processable OLEDs, we employed a sensitization strategy by utilizing high‐performance TADF materials featuring rapid RISC rates as sensitizers, which improved device efficiency and suppressed efficiency roll‐off.^[^ ^35^, ^36^, ^37^, ^38^ ^]^ The device's architecture consisted of: ITO/ PEDOT:PSS:PFI (60 nm)/ mCP: 28 wt% 4tCz‐PhCz: 2 wt% emitters (40 nm)/ TSPO1 (5 nm)/ TmPyPB (50 nm)/Liq (2 nm)/Al (100 nm) (Figure 3a). In contrast to the non‐sensitized devices, the present architecture utilizes mCP, TSPO1, TmPyPB, and Liq as the host matrix, hole‐blocking layer, electron‐transport layer, and electron‐injection layer, respectively. The sensitizer 4tCz‐PhCz with a concentration of 28 wt% was selected based on its exceptional PLQY, rapid *k*_RISC_, and optimal spectral overlap between its PL spectrum and the absorption spectra of DBN‐Pym/ DBN‐PhPym (Figure S23, Supporting Information).^[^ ^39^ ^]^ The corresponding molecular structures are depicted in Figure S24 (Supporting Information). The EL emission characteristics of both emitters—peaking at 526 nm (FWHM: 32 nm) for DBN‐Pym and 521 nm (FWHM: 34 nm) for DBN‐PhPym—showed excellent agreement with their corresponding PL profiles and non‐sensitized device performance (Figure 3b). This spectral consistency confirms that the sensitization process preserves the intrinsic emission properties of the emitters. Furthermore, the devices achieved CIE coordinates of (0.256, 0.690) for DBN‐Pym and (0.213, 0.693) for DBN‐PhPym, representing exceptional green color purity that meets the stringent requirements for display applications. Both sensitized devices exhibited exceptional spectral stability, which remained nearly unchanged as the driving voltage increased (Figure S25, Supporting Information). The *J*–*V*–*L* curves, EQE‐*L* curves, CE–*L*, and PE–*L* curves are depicted in Figure 3c–e, and the corresponding data are listed in **Table** **2**. The sensitized DBN‐Pym device exhibited significantly enhanced performance, with a maximum EQE (EQE_max_) of 19.9%, a maximum current efficiency (CE_max_) of 78.1 cd A^−1^, and a maximum power efficiency (PE_max_) of 42.2 lm W^−1^, underscoring a substantial improvement over its non‐sensitized counterpart. Notably, the sensitized device maintained high EQE values of 19.8% at 1000 cd m^−2^ and 16.7% even at 10 000 cd m^−2^, demonstrating remarkable stability under high luminance conditions. The sensitized DBN‐PhPym device delivered even more exceptional performance, achieving an EQE_max_ of 29.0%, a CE_max_ of 105.4 cd A^−1^, and a PE_max_ of 74.0 lm W^−1^. It also displayed outstanding efficiency roll‐off characteristics, retaining EQEs of 28.3% at 1000 cd m^−2^ and 20.7% at 10 000 cd m^−2^. These results firmly validate the efficacy of the sensitization strategy in mitigating efficiency roll‐off at high brightness levels.^[^ ^40^ ^]^ The angle‐resolved emission experiment revealed a closely Lambertian distribution of light from the sensitized device (Figure S26, Supporting Information), validating the accuracy of the estimated EQE values. Compared to DBN‐Pym, the derivative DBN‐PhPym demonstrated significantly improved device performance. This enhancement primarily stems from its more twisted conformational geometry, which effectively mitigates ACQ by restricting detrimental intermolecular interactions and suppresses the Dexter energy transfer process due to an enlarged intermolecular distance. Moreover, DBN‐PhPym also exhibits a shorter excited‐state lifetime, which further contributes to enhanced performance by diminishing exciton annihilation phenomena under operational conditions. For comparison, a sensitized device was also fabricated using the compound SBN‐PhPym with the same device architecture. It exhibited inferior performance compared to DBN‐PhPym, likely due to its poorer solubility, as indicated by the corresponding data in Figure S27 and Table S3 (Supporting Information). Furthermore, the markedly lower efficiency of the mCP‐based non‐sensitized control device of DBN‐Pym and DBN‐PhPym compared to the sensitized device provides compelling evidence that the efficiency enhancement is primarily attributable to the sensitization mechanism itself. This controlled comparison effectively rules out the host material as the primary source of improvement, thereby strengthening the conclusion regarding the efficacy of the proposed sensitization strategy. The corresponding performance of these non‐sensitized devices is presented in Figure S28 and Table S4 (Supporting Information). Notably, these devices exhibited a high turn‐on voltage, along with significantly reduced EL efficiency and luminance, with the maximum luminance rapidly dropping below 1000 cd m^−2^. The unsatisfactory device performance further reinforces the importance of the sensitization strategy. The outstanding performance of the sensitized devices ranks among the best results for solution‐processed narrowband OLEDs based on MR‐TADF small molecules (Figure 3e; Figure S29 and Table S5, Supporting Information).^[^ ^41^, ^42^, ^43^, ^44^, ^45^, ^46^, ^47^, ^48^, ^49^, ^50^ ^]^ This achievement underscores the potential of the acceptor‐bridging engineering strategy, which employs covalently bridged multiple MR‐TADF units to develop solution‐processible emitters, paving the way for high‐performance devices with outstanding efficiency and narrowband emission.

**Table 2:** Summary of the EL data of the sensitized devices based on DBN‐Pym and DBN‐PhPym.

Emitter	*λ*_em_ [nm]^a)^	FWHM [nm]^a)^	V_on_ [V]^b)^	CE_max_ [cd A^−1^]	PE_max_ [lm W^−1^]	EQE [%]^c)^	CIE [x,y]^a)^
DBN‐Pym	526	32	3.3	78.1	42.2	19.9/19.8/16.7	(0.256, 0.690)
DBN‐PhPym	521	34	3.0	105.4	74.0	29.0/28.3/20.7	(0.213, 0.693)

^a)^ EL spectra measured at 100 cd m^−2^.

^b)^ Turn‐on voltage at 1 cd m^−2^.

^c)^ Maximum external quantum efficiency, and values at 1000 and 10 000 cd m^−2^, respectively.

## 3 Conclusion

In summary, this work establishes a robust molecular design strategy for developing solution‐processable, narrowband green MR‐TADF emitters through acceptor‐bridge engineering. By integrating multiple MR‐building blocks with electron‐withdrawing bridging units, we simultaneously enhanced solution processability, suppressed aggregation‐induced spectral broadening, and precisely tuned emission characteristics, thereby achieving an optimal balance between color purity and device performance. The developed emitters DBN‐Pym and DBN‐PhPym exhibit pure green emission with peaks at 514 and 513 nm, respectively, accompanied by narrow FWHM values of 24 and 30 nm. When incorporated into sensitized solution‐processed OLEDs, DBN‐PhPym delivered outstanding performance with an EQE_max_ of 29.0 %. Notably, it maintains high EQE values of 28.3 % at 1000 cd m^−2^ and 20.7% even at 10 000 cd m^−2^. The emitter also exhibits an FWHM of 34 nm and CIE coordinates of (0.213, 0.693), representing state‐of‐the‐art performance among solution‐processed MR‐TADF‐based OLEDs. These results validate the proposed molecular design as a promising platform for developing high‐efficiency, narrowband emitters compatible with solution‐processing techniques.

## Conflict of Interest

The authors declare no conflict of interest.

## Supporting information



Supporting Information

## Data Availability

The data that support the findings of this study are available in the supplementary material of this article.
