# Supplementary material for: Acceptor‐Bridged Engineering Enables Highly Efficient Solution‐Processed Pure‐Green MR‐TADF OLEDs With External Quantum Efficiency of 29% and Small Roll‐Off
Source: Adv Sci (Weinh). 2025 Nov 11;13(6):e18308. doi: 10.1002/advs.202518308 (PMC12866874; doi:10.1002/advs.202518308)
Supplement: Supplementary file 1 — Supporting Information [file ADVS-13-e18308-s001.docx]

Supporting Information

**Acceptor-Bridged Engineering Enables Highly Efficient Solution-Processed Pure-Green MR-TADF OLEDs with External Quantum Efficiency of 29% and Small Roll-Off**

*Xuming Zhuang, Qing Zhang,* *Jinbei Wei*, Zhiqiang Li, Baoyan Liang, Chenguang Wang, Hai Bi*, Yue Wang, and Geyu Lu**

Dr. X. Zhuang, Dr. Z. Li, Dr. B. Liang, Prof. H. Bi, Prof. Y. Wang

Jihua Laboratory, 28 Huandao South Road, Foshan 528200, Guangdong Province, China.

Q. Zhang, Prof. J. Wei, Prof. C. Wang, Prof. G. Lu

State Key Laboratory of Integrated Optoelectronics, JLU Region, Key Laboratory of Advanced Gas Sensors of Jilin Province, College of Electronic Science and Engineering, Jilin University, Changchun, 130012, China

Prof. Y. Wang

State Key Laboratory of Supramolecular Structure and Materials, College of Chemistry, Jilin University, Changchun 130012, China

E-mail: jinbwei@jlu.edu.cn (J. W), bihai@jihualab.ac.cn (H. Bi), lugy@jlu.edu.cn (G. Lu)

**Table of Contents**

[**1.** **General Information** 3](#_Toc149806676)

[**2. Theoretical calculation** 3](#_Toc149806677)

[**3.** **Calculation Equations of External Quantum Efficiency and Formulas for the Photophysical Parameters** 4](#_Toc149806678)

[**4.** **Device Fabrication and Measurements** 5](#_Toc149806679)

[**5.** **Synthesis of Materials** 6](#_Toc149806680)

[**6.** **Supplementary Figures** 8](#_Toc149806681)

[**7.** **Supplementary Tables** 20](#_Toc149806682)

[**8.** **References** 24](#_Toc149806683)

1. **General Information**

A Bruker Autoflex Speed mass spectrometer was employed to measure the mass spectra. Bruker AVANCE III 400 MHz spectrometers were used to measure the ¹H and ¹³C NMR spectra, respectively, with tetramethylsilane (TMS) as the internal standard. A Shimadzu RF–5301 PC spectrometer and a Shimadzu UV–2550 spectrophotometer were used to record the PL emission spectra and UV–Vis absorption spectra, respectively. The phosphorescence spectra taken at liquid nitrogen temperature (77 K) were recorded by Ocean Optics QE Pro with a 365 nm Ocean Optics LLS excitation source. The photoluminescence quantum yields (PLQYs) of the solutions and organic films were detected from a Hamamatsu Absolute PL C11347-11 quantum yield spectrometer. The FLS1000 fluorescence lifetime measurement system was selected to investigate the transient PL decay. In the range of 25 to 800 °C, TA Q500 thermogravimeter was selected to perform the thermogravimetric analysis (TGA) under nitrogen atmosphere at a heating rate of 10 K min^–1^. The BAS 100W Bioanalytical electrochemical workstation was used to measure the electrochemical properties with a platinum disk as the working electrode, a platinum wire as the auxiliary electrode, a porous glass wick with Ag/Ag^+^ as the pseudo-reference electrode, and ferrocene/ferrocenium as the internal standard. And 0.1 M solution of *n*-Bu_4_NPF_6,_ which was the supporting electrolyte, was utilized to measure the oxidation (in anhydrous dichloromethane) or reduction (in anhydrous tetrahydrofuran) potentials at a scan rate of 100 mV s^–1^. Atomic force microscopic (AFM) images were recorded on a Bruker nanooptics AFM. Angle-resolved and polarization-resolved PL measurements were performed using an R1-OLED system (ideaoptics Co., Ltd). The thicknesses of the spin-coated films were measured using a step profiler (KLA Tencor P-7).

1. **Theoretical calculation**

The ground state geometries of gas state were fully optimized by B3LYP method including Grimme’s dispersion correction with 6-31G(d, p) basis set using Gaussian 16 software package. HOMO and LUMO were visualized with Gaussview 6.0. ^[S1–S5]^ The NTO analysis was performaned using Multiwfn package.^[S6]^

1. **Calculation Equations of External Quantum Efficiency and Formulas for the Photophysical Parameters**

When assuming the external emission profile is Lambertian distribution, the EQE can be determined by the following equations:

$\mathrm{EQE}=\frac{N_{p}}{N_{e}}$ **(S1)**

$N_{p}=\frac{\int L_{e}(\lambda)\cdot W(\lambda)\cdot d\lambda}{h\times c}\times\pi\times D$  **(S2)**

$N_{e}=\frac{I}{e}$ **(S3)**

Where *N_p_* is the photons number, *N_e_* is the electrons number, *L_e_* (λ) is the spectral radiance (W sr^-1^ m^-2^ nm^-1^), *W* (λ) is the wavelength, *d*λ=1, *D* is the emitting area, *h* is the Planck’s constant, *c* is the speed of light in vacuum, *e* is the elementary charge of electron and *I* is the injected current.

The calculation of the kinetic parameters assumes that internal conversion process of the singlet exciton is the main nonradiative decay.^[S7-S9]^

*k*_r_ = *Φ*_F_*k*_F_ + *Φ*_TADF_*k*_TADF_ (**S4**)

*k*_nr_ = *k*_r_ (1-*Φ*_PL_)/*Φ*_PL_ (**S5**)

*k*_ISC_= *k*_F_ - *k*_r_ - *k*_nr_ (**S6**)

*k*_RISC_= (*k*_F_*k*_TADF_*Φ*_TADF_)/(*k*_ISC_*Φ*_F_) (**S7**)

Where *k*_F_ and *k*_TADF_ represent the decay rate constants for prompt and delayed fluorescence, respectively, which are in reciprocal relationship with the decay time constants (*τ*_F_ and *τ*_TADF_) experimentally determined from transient PL characteristics; *Φ*_PL_ is the total fluorescence quantum yield, *Φ*_F_ is the prompt fluorescent component of *Φ*_PL_, *Φ*_TADF_ is the delayed fluorescent component of *Φ*_PL_. *k*_r_, *k*_nr_, *k*_ISC_ and *k*_RISC_ are rate constants of radiative decay, non-radiative decay, intersystem crossing and reverse intersystem crossing, respectively.

1. **Device Fabrication and Measurements**

The indium tin oxide (ITO) glass substrates with a sheet resistance of 35 Ω per square were cleaned with optical detergent, deionized water, acetone and isopropanol successively. After UV-ozone treatment for 15 min, a (PEDOT:PSS):PFI mixture with a mass ratio of 1:5 was used as hole-injection layer and directly spin-coated on the ITO substrate at the spin speed of 3500 rpm for 40 s, and dried at 180 °C for 15 min. Then the glasses were transformed into a N_2_ atmosphere glovebox. For the emissive layer, the constituent materials were dissolved in chlorobenzene at a concentration of 15 mg/ml (SBN-PhPym at a concentration of 1 mg/ml). The emissive layer was also prepared by spin-coating with 1500 rpm for 30 s directly on the hole-injection layer and annealed at 80 °C for 60 min. Finally, the ITO-coated glasses are transferred to a vacuum deposition system and the DMFBD-TRZ, ETL-2:Liq (1:1), Liq and Al layers (non-sensitized devices) or TSPO1, TmPyPB, Liq and Al layers (sensitized devices) were consecutively thermally evaporated onto the emissive layer in a vacuum chamber of < 9 × 10^−5^ Pa. All organic materials used in the devices, with a purity greater than 99%, were purchased from Changchun Tuocai Technology Co., Ltd. and Xi'an Yuri Solar Co., Ltd. The EL spectrum, CIE coordinate and luminance intensity of the OLEDs were recorded by Konica Minolta CS2000, meanwhile, the current density (J) and driving voltage (V) were recorded by Keithley 2400. By assuming Lambertian distribution, the external quantum efficiency (EQE) was estimated according to brightness, electroluminescence spectrum and current density. The electroluminescence pictures were obtained by using fluorescence microscopy.

1. **Synthesis of Materials**

All reagents were purchased from Energy Chemical Co. and Bide Pharmatech Ltd. and were used immediately without further purification. The Schlenk technique was strictly performed under nitrogen conditions in all reactions, and the detailed synthetic procedures are shown below.

**Scheme S1**. Synthetic procedure of DBN-Pym, DBN-PhPym and SBN-PhPym.

***Synthesis of DBN-Pym:*** BNCz-Bpin (1.69 g, 2.20 mmol), 4,6-Dichloropyrimidine (0.15 g, 1.00 mmol), potassium carbonate (K_2_CO_3_) (0.61 g, 4.40 mmol) were added with water (15 ml) and 1,4-dioxane (60 ml). The mixture was bubbled with nitrogen for 5 minutes, and tetrakis(triphenylphosphine)palladium(0) (Pd(PPh_3_)_4_) (0.12 g, 0.10 mmol) was added under a high flow of nitrogen. The mixture was then heated to reflux and stirred for 12 hours. After cooling to room temperature, the reaction mixture was extracted with dichloromethane and water, and the combined organic layer was condensed under vacuum. The crude product was then further purified by column chromatography using a mixture eluent of petroleum ether/dichloromethane (4:1) to afford a yellow solid (1.24 g). Yield: 91%. ^1^H NMR (400 MHz, Chloroform-*d*) δ 9.75 (s, 1H), 9.12 (s, 8H), 8.72 (s, 1H), 8.65 (d, *J* = 8.8 Hz, 4H), 8.48 (s, 4H), 8.26 (s, 4H), 7.63 (d, *J* = 8.7 Hz, 4H), 1.68 (s, 36H), 1.44 (s, 36H). ^13^C NMR (101 MHz, Chloroform-*d*) δ 164.96, 159.37, 145.35, 144.64, 144.48, 141.59, 140.88, 138.23, 129.62, 126.99, 124.74, 124.46, 123.69, 121.57, 120.79, 117.44, 114.55, 114.01, 106.60, 35.11, 34.72, 32.14, 31.76. MALDI-TOF (m/z) calcd for C_96_H_98_B_2_N_6_ [M+H]^+^:1356.804; Found: 1356.504.

***Synthesis of DBN-PhPym:*** BNCz-Bpin (1.69 g, 2.20 mmol), 4,6-Dichloro-2,5-diphenylpyrimidine (0.30 g, 1.00 mmol), potassium carbonate (K_2_CO_3_) (0.61 g, 4.40 mmol) were added with water (15 ml) and 1,4-dioxane (60 ml). The mixture was bubbled with nitrogen for 5 minutes, and tetrakis(triphenylphosphine)palladium(0) (Pd(PPh_3_)_4_) (0.12 g, 0.10 mmol) was added under a high flow of nitrogen. The mixture was then heated to reflux and stirred for 12 hours. After cooling to room temperature, the reaction mixture was extracted with dichloromethane and water. The combined organic layer was condensed under vacuum. Then the crude product was further purified by column chromatography with a mixture eluent of petroleum/dichloromethane ether (4:1) to afford the yellow solid (0.76 g). Yield: 50%. ^1^H NMR (400 MHz, Methylene Chloride-*d*_2_) δ 9.17 (d, *J* = 1.9 Hz, 4H), 8.86 (dd, *J* = 6.7, 3.0 Hz, 2H), 8.57 – 8.54 (m, 8H), 8.29 (d, *J* = 2.1 Hz, 4H), 7.92 (d, *J* = 8.9 Hz, 4H), 7.76 – 7.70 (m, 2H), 7.59 (d, *J* = 1.4 Hz, 1H), 7.58 (d, *J* = 2.4 Hz, 2H), 7.56 (d, J = 2.1 Hz, 2H), 7.53 (d, J = 2.1 Hz, 2H), 7.34 (t, *J* = 7.8 Hz, 2H), 7.05 (t, *J* = 7.5 Hz, 1H), 1.69 (s, 36H), 1.48 (s, 36H). ^13^C NMR (101 MHz, Methylene Chloride-*d*_2_) δ 166.71, 146.08, 145.46, 144.18, 144.04, 142.01, 138.52, 137.94, 136.68, 132.13, 131.45, 130.27, 130.23, 129.46, 129.17, 128.94, 128.70, 127.23, 124.83, 124.12, 123.44, 121.93, 121.55, 117.74, 114.59, 110.49, 35.54, 35.07, 32.32, 31.89. MALDI-TOF (m/z) calcd for C_108_H_106_B_2_N_6_ [M+H]^+^:1508.867; Found: 1508.829.

***Synthesis of SBN-PhPym:*** BNCz-Bpin (0.84 g, 1.10 mmol), 4-Chloro-2,6-diphenylpyrimidine (0.27 g, 1.00 mmol), potassium carbonate (K_2_CO_3_) (0.44 g, 3.00 mmol) were added with water (15 ml) and 1,4-dioxane (60 ml). The mixture was bubbled with nitrogen for 5 minutes, and tetrakis(triphenylphosphine)palladium(0) (Pd(PPh_3_)_4_) (0.12 g, 0.10 mmol) was added under a high flow of nitrogen. The mixture was then heated to reflux and stirred for 12 hours. After cooling to room temperature, the reaction mixture was extracted with dichloromethane and water. The combined organic layer was condensed under vacuum. Then the crude product was slurried with dichloromethane and filtered to afford the yellow solid (0.81 g). Yield: 93%. ^1^H NMR (400 MHz, Chloroform-*d*) δ 9.05 (s, 2H), 8.92 (d, *J* = 1.9 Hz, 2H), 8.81 (dd, *J* = 6.7, 3.0 Hz, 2H), 8.41 (d, *J* = 8.8 Hz, 2H), 8.35 (d, *J* = 1.7 Hz, 2H), 8.34 – 8.30 (m, 2H), 8.13 (d, *J* = 2.0 Hz, 2H), 7.90 (s, 1H), 7.66 (d, *J* = 7.0 Hz, 3H), 7.61 (d, *J* = 2.1 Hz, 1H), 7.59 (dd, *J* = 4.1, 2.4 Hz, 4H), 1.66 (s, 18H), 1.53 (s, 18H). MALDI-TOF (m/z) calcd for C_62_H_59_BN_4_ [M+H]^+^:870.483; Found: 871.720.

1. **Supplementary Figures
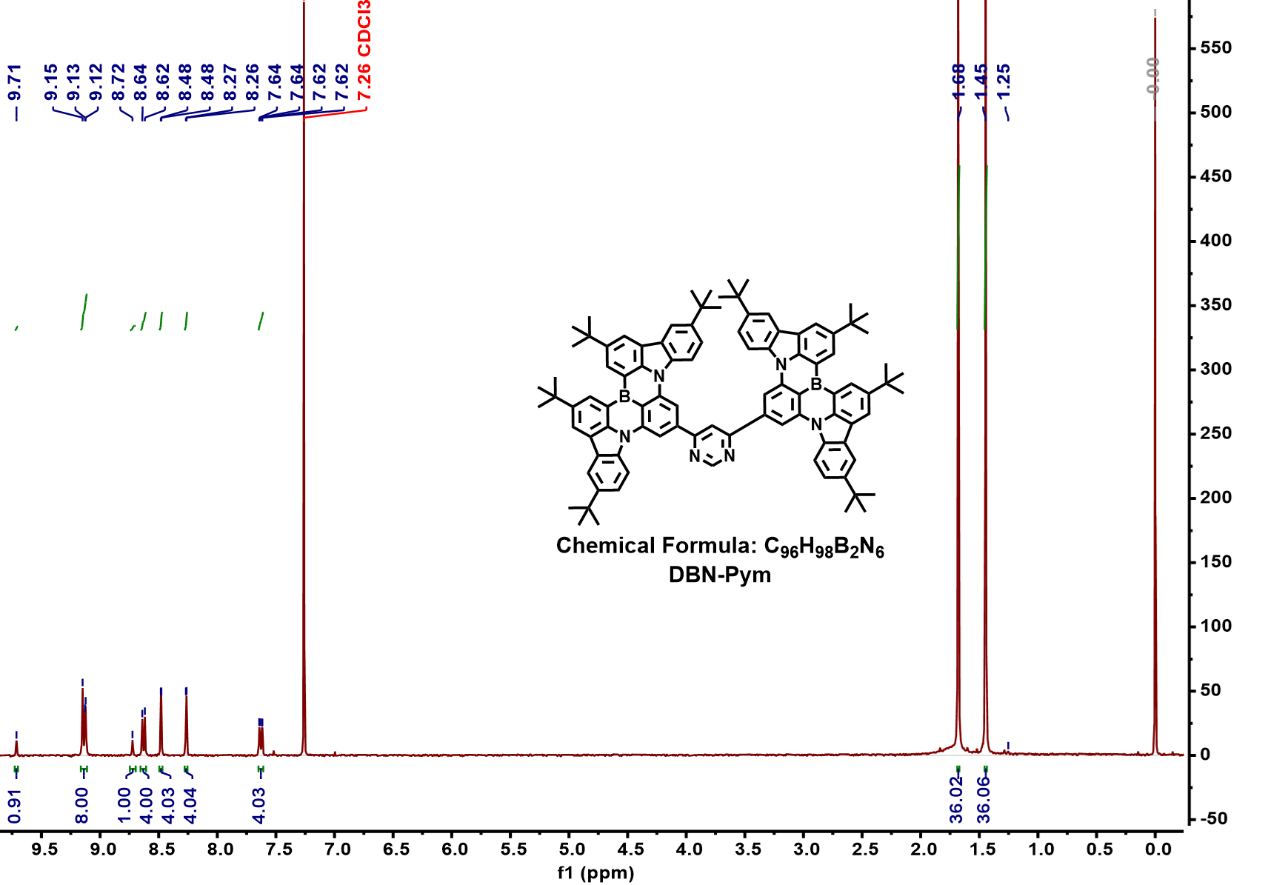
**

**Figure S1.** ^1^H NMR spectrum of DBN-Pym in Chloroform-*d*.


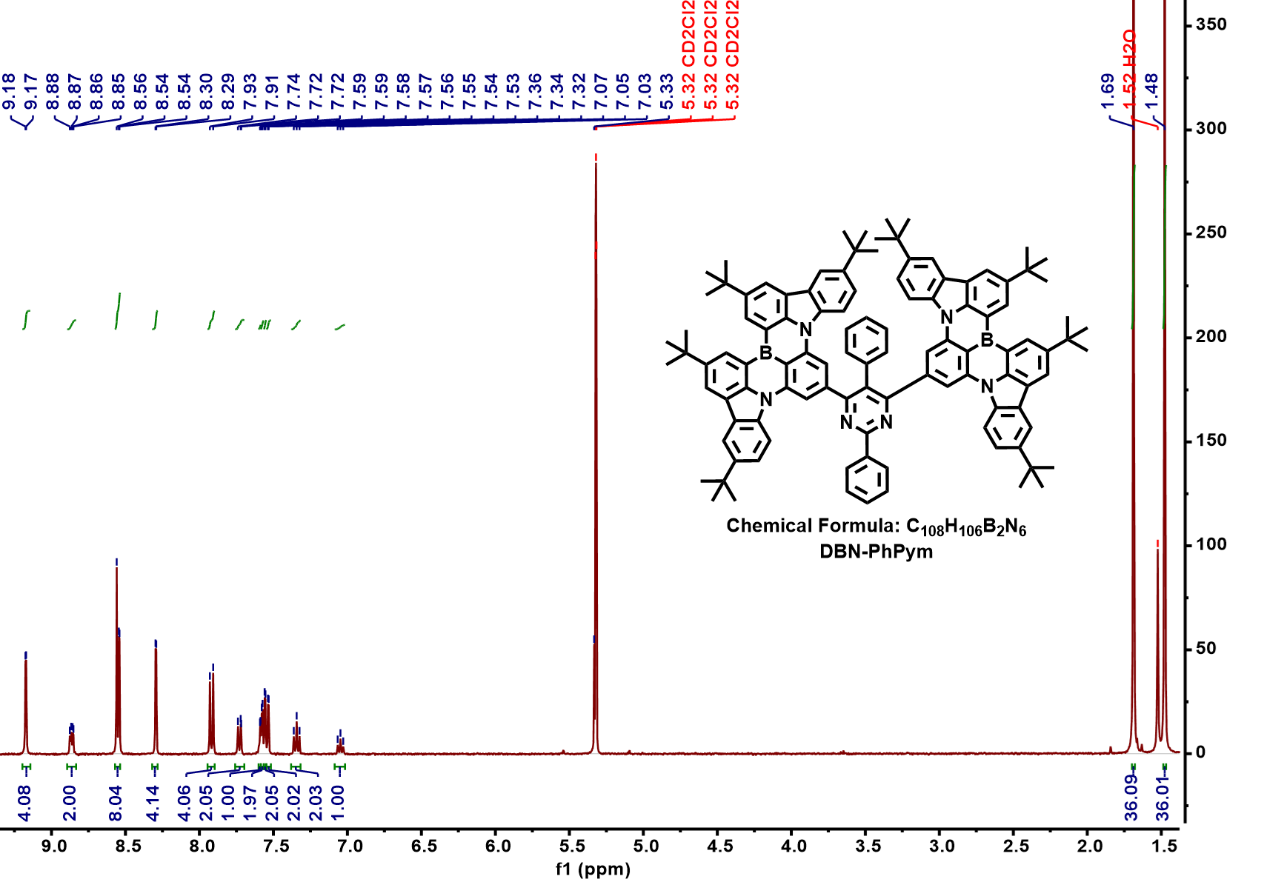


**Figure S2.** ^1^H NMR spectrum of DBN-PyPym in Methylene Chloride-*d*_2_.


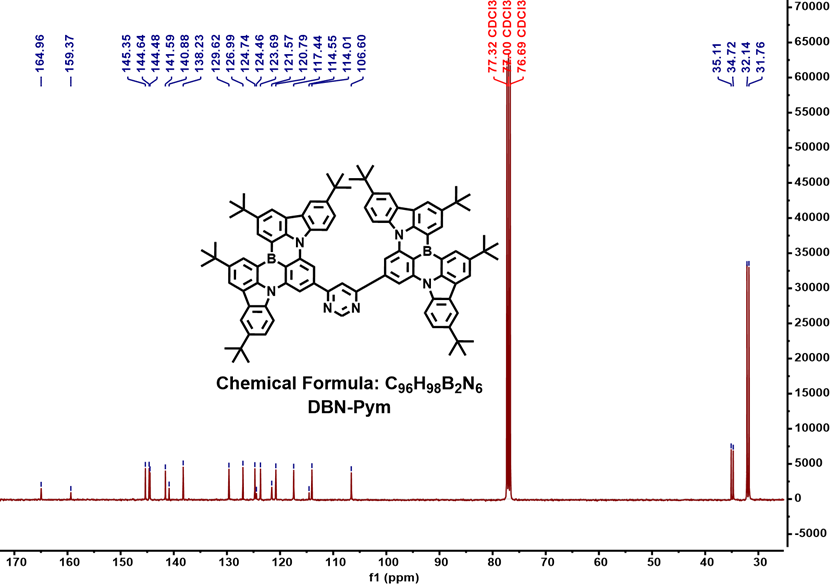


**Figure S3.** ^13^C NMR spectrum of DBN-Pym in Chloroform-*d*.


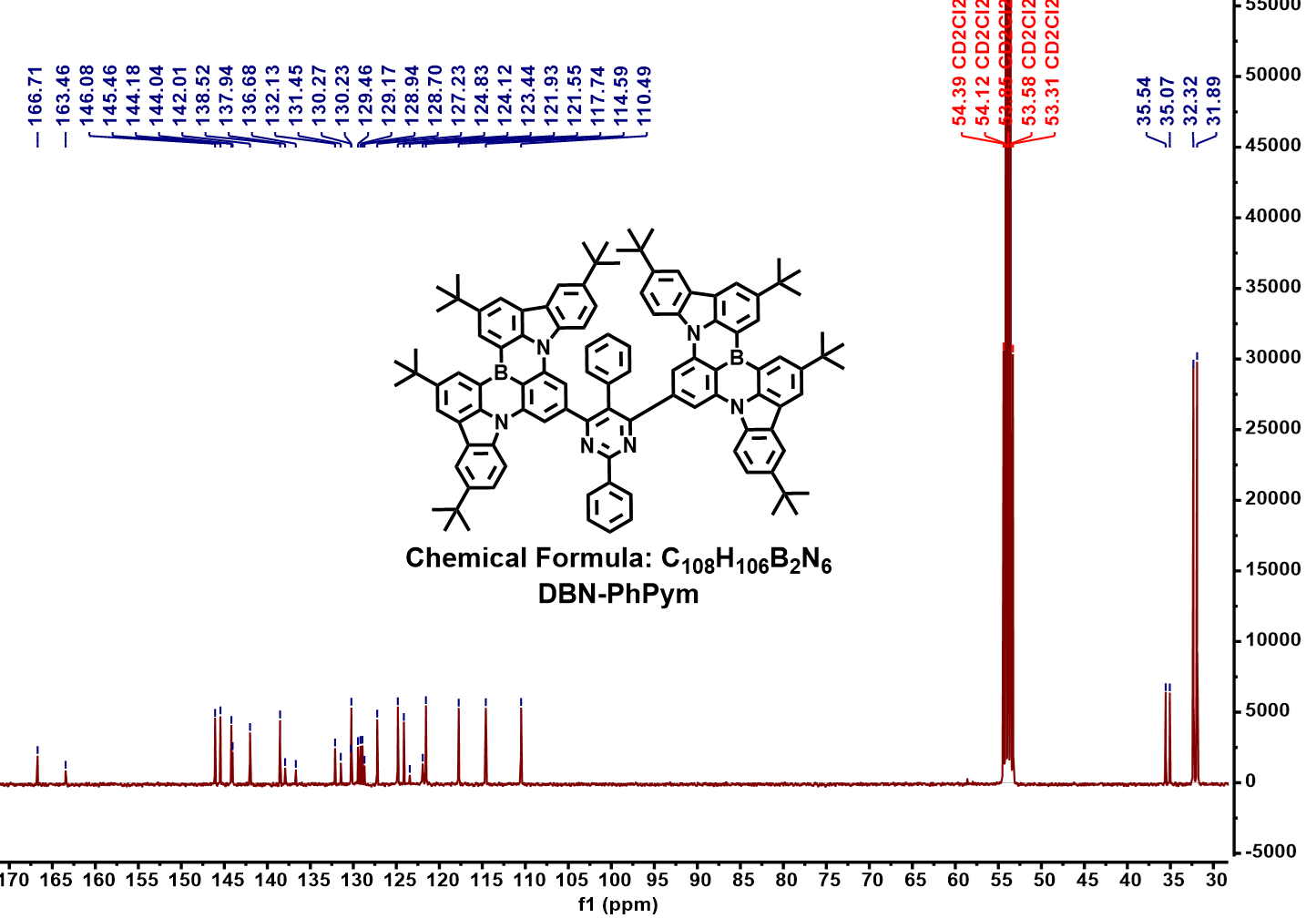


**Figure S4.** ^13^C NMR spectrum of DBN-PhPym in Methylene Chloride-*d*_2_.


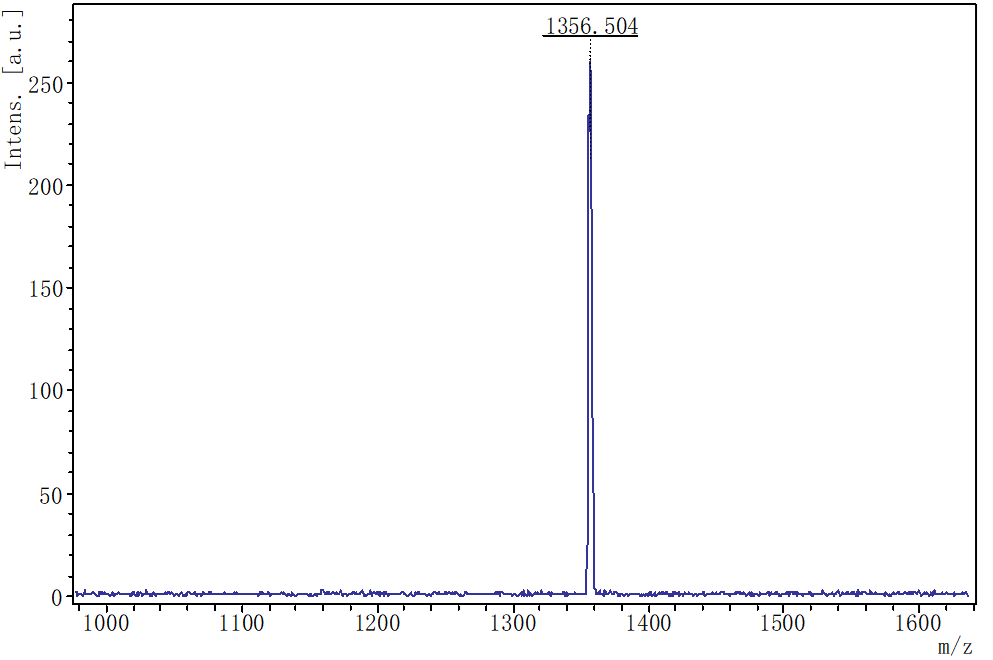


**Figure S5.** MALDI-TOF-MS of DBN-Pym.


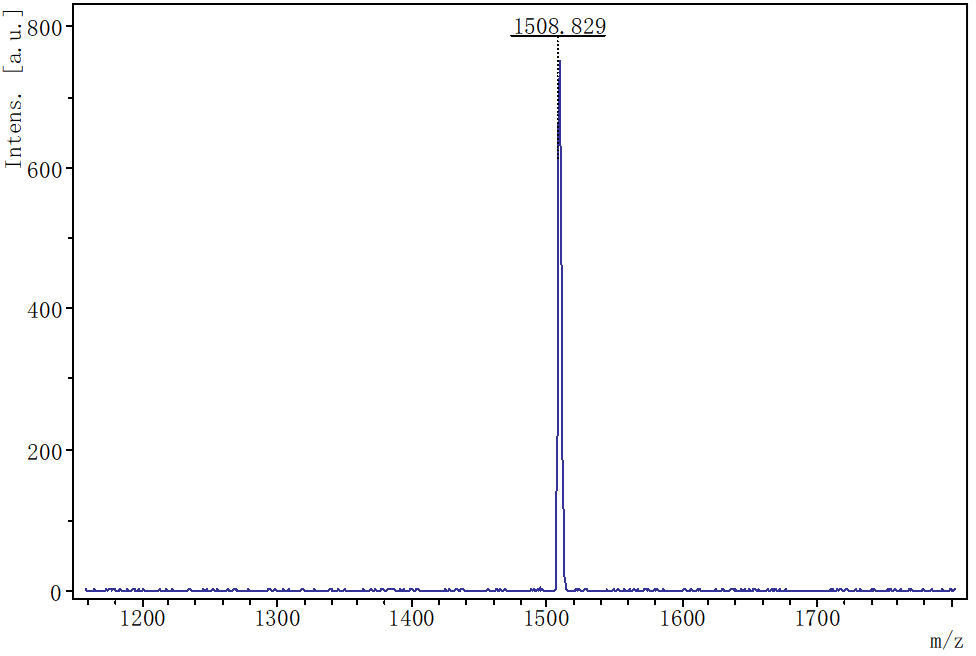


**Figure S6**. MALDI-TOF-MS of DBN-PhPym.

**
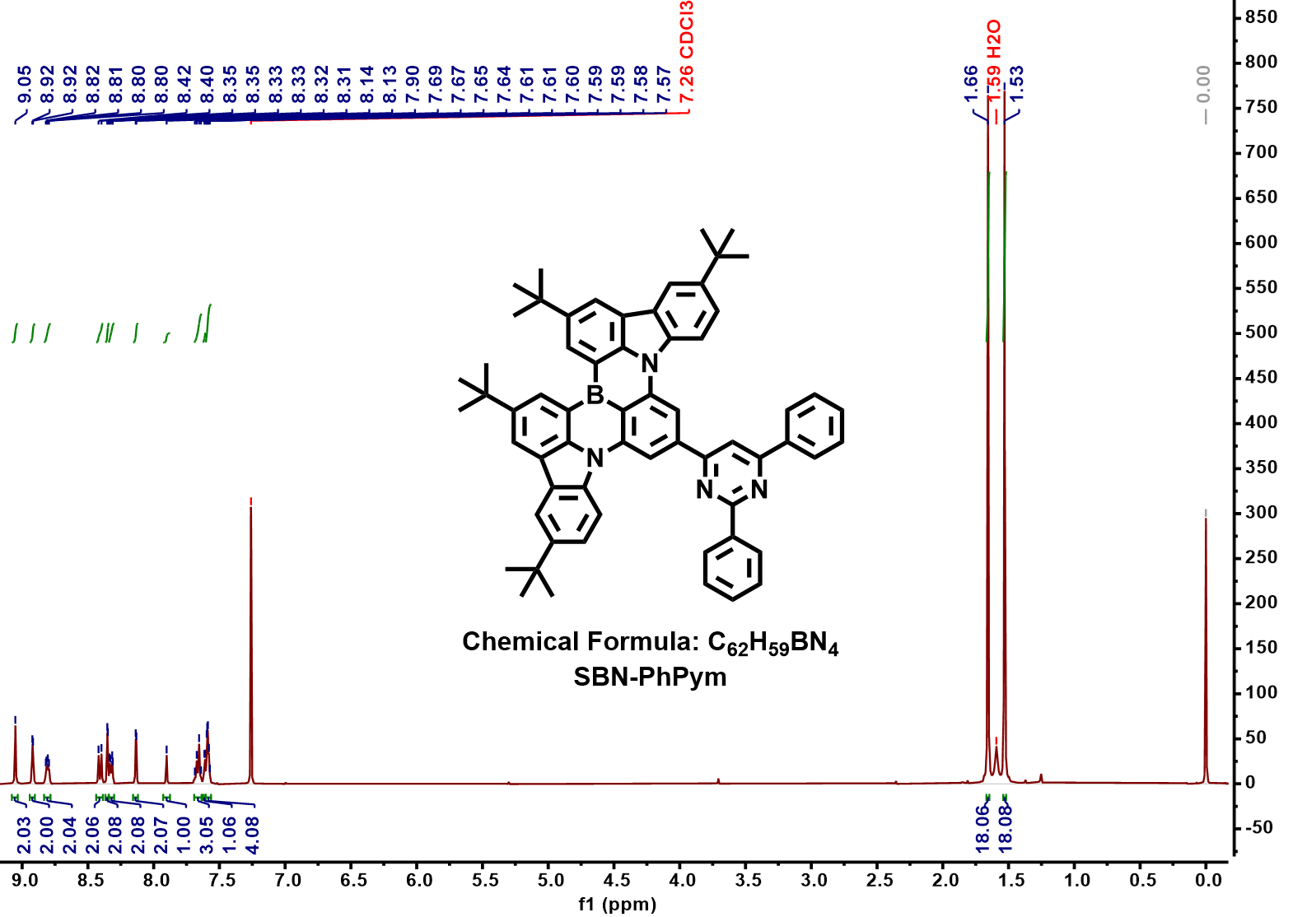
**

**Figure S7.** ^1^H NMR spectrum of SBN-PhPym in Methylene Chloroform-*d*.

**Figure S8**. MALDI-TOF-MS of SBN-PhPym.

**Figure S9.** (a) TGA curve and (b) DSC curve of DBN-Pym and DBN-PhPym.

**Figure S10.** CV curves of DBN-Pym and DBN-PhPym**.**

**
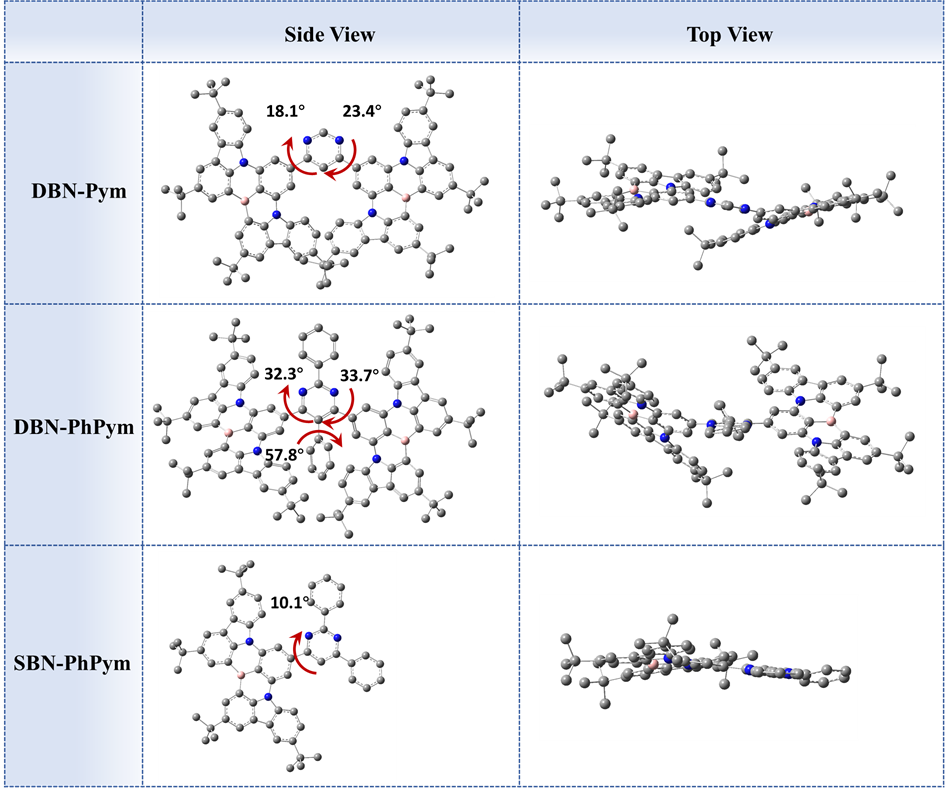
**

**Figure S11.** Optimized ground state (S_0_) structure of DBN-Pym and DBN-PhPym.


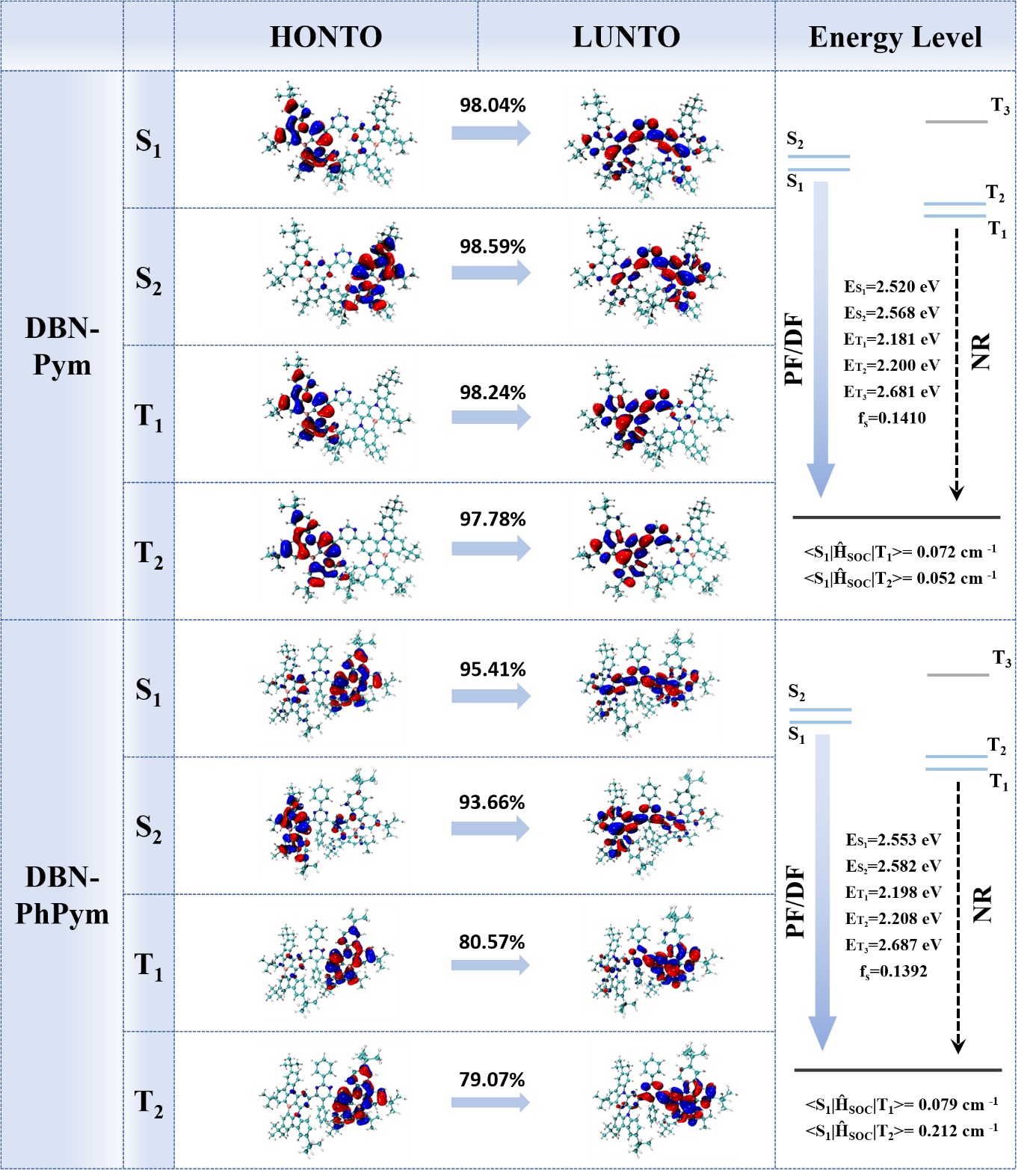


**Figure S12.** Energy-level diagrams and related NTOs for the singlet and triplet excited states of DBN-Pym and DBN-PhPym. Transition energies for S_1_, S_2_, T_1_ and T_2_, and the SOC matrix elements were calculated at the B3LYP/6-31G(d) levels of theory, respectively.

**Figure S13.** AFM image of DBN-Pym, DBN-PhPym, BNCz, SBN-PhPym and BNCz doped films in mCP host matrix with doping concentration of 2.0 wt%.

**Figure S14.** Ultraviolet-visible (UV-Vis) absorption, fluorescence (298K) and phosphorescence (77K) spectra of SBN-PhPym in dilute toluene solution.

**Figure S15.** Photograph of BNCz, DBN-Pym, DBN-PhPym and SBN-PhPym dissolve in chlorobenzene.

**Figure S16.** Angle-dependent p-polarized PL intensity of the doped films (DBN-Pym/DBN-PhPym doped in mCP host matrix at a 2 wt% doping concentration). (a) DBN-Pym based film, (b) DBN-PhPym based film. (Θ// = 67% denotes isotropic emitting dipole orientation, Θ// = 100% denotes completely horizontal emitting dipole orientation)

**Figure S17.** PL spectra of DBN-Pym and DBN-PhPym in mCP host matrix at different doping concentrations

**Figure S18.** (a), (c) Prompt decay in nanosecond scales and (b), (d) Delayed decay in millisecond scales curves of DBN-Pym and DBN-PhPym in mCP host matrix at different doping concentrations.


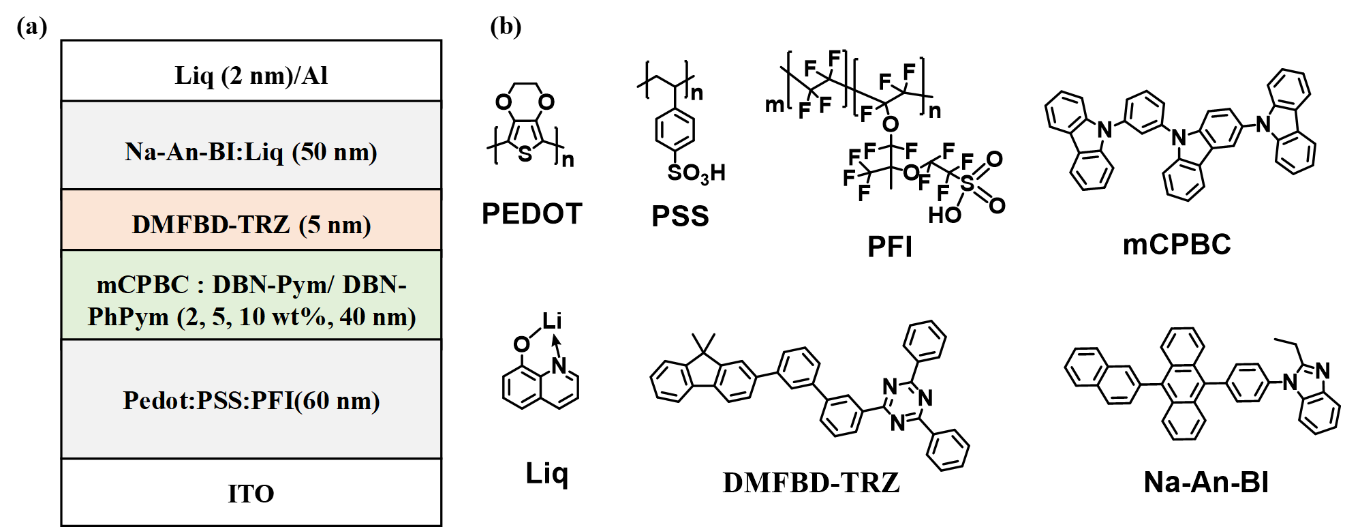


**Figure S19.** (a) Device configuration, (b) Molecular structures of materials used in the devices.


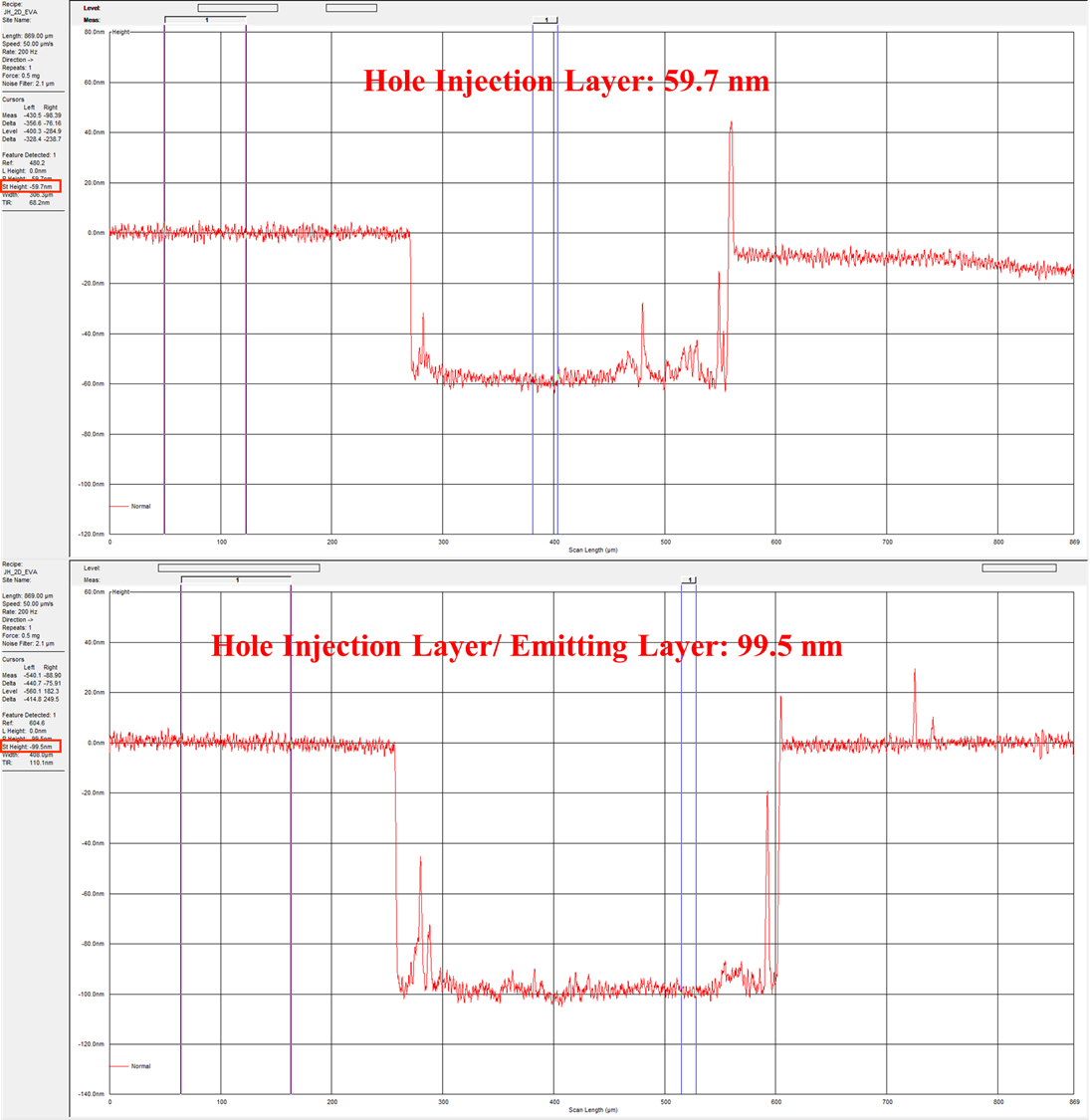


**Figure S20.** The thicknesses of spin-coated films measured using a step profiler. (a) The thickness of hole injection layer, (b) the total thickness of hole inhection layer/emitting layer.

**Figure S21.** EL characteristics of the DBN-Pym based devices. (a) EL spectra, (b) EQE-*L* curves, (c) *J*-*V*-*L* curves and (d) CE-*L* and PE-*L* curves.

**Figure S22.** EL characteristics of the DBN-PhPym based devices. (a) EL spectra, (b) EQE-*L* curves, (c) *J*-*V*-*L* curves and (d) CE-*L* and PE-*L* curves.

**Figure S23**. UV–vis absorption spectrum of DBN-Pym and DBN-PhPym in toluene at a concentration of 10^−5^ M and PL spectrum of 30 wt% 4tCz-PhCz in mCP host matrix film.

**Figure S24.** Molecular structures of materials used in the sensitized devices.

**Figure S25.** EL Spectra of the sensitized devices under different driving voltages. (a) DBN-Pym-based device, (b) DBN-PhPym-based device.

**Figure S26.** Angel-dependent EL intensity of the sensitized device. (a) DBN-Pym-based device, (b) DBN-PhPym-based device.

**Figure S27.** EL characteristics of the sensitized devices based on SBN-PhPym with 2 wt% doping concentration. (a) EL spectra, (b) EQE-*L* curves, (c) *J*-*V*-*L* curves and (d) CE-*L* and PE-*L* curves.

**Figure S28.** EL characteristics of the mCP-host non-sensitized devices with 2 wt% doping concentration. (a) EL spectra, (b) EQE-*L* curves, (c) *J*-*V*-*L* curves and (d) CE-*L* and PE-*L* curves.

**Figure S29**. Device performance comparison graph of the EQE value at operational intensity of 1000 cd/m^2^ of MR-TADF based solution-processed OLEDs.


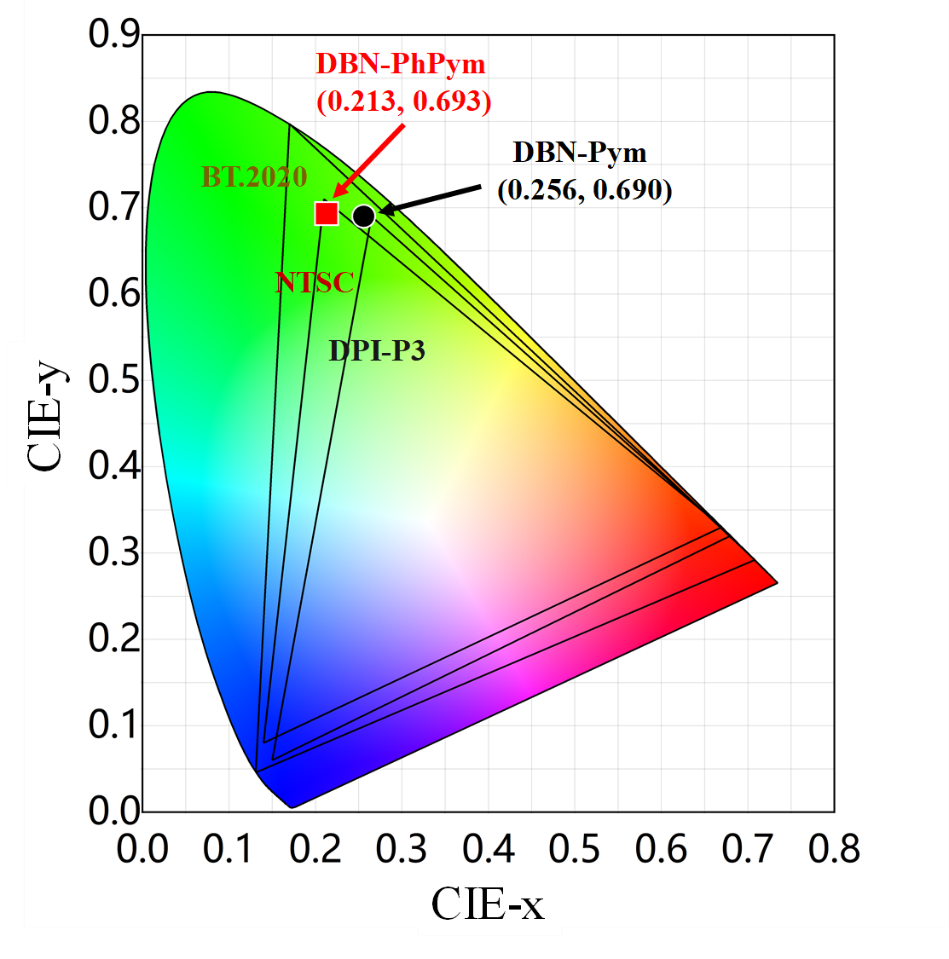


**Figure S30**. CIE plot of the sensitized sOLEDs based on DBN-Pym and DBN-PhPym.

1. **Supplementary Tables**

**Table S1.** Summary of photophysical data of 2 wt % emitter: mCPBC (doped films without sensitizer).

| Emitter | *λ*_em_^a)^  [nm] | FWHM^b)^  [nm] | *Φ*_PL_^c)^  [%] | *Φ*_F_^d)^  [%] | *Φ*_TADF_^e^  [%] | *τ*_F_^f)^  [ns] | *τ*_TADF_^g)^  [μs] | *k*_F_^h)^  [10^7^ s^−1^] | *k*_IC_^i)^  [10^7^ s^−1^] | *k*_ISC_^j)^  [10^7^ s^−1^] | *k*_TADF_^k)^  [10^4^ s^−1^] | *k*_RISC_^l)^  [10^4^ s^−1^] |
| --- | --- | --- | --- | --- | --- | --- | --- | --- | --- | --- | --- | --- |
| DBN-Pym | 525 | 34 | 75.0 | 69.8 | 5.2 | 8.7 | 53.3 | 8.0 | 2.7 | 0.8 | 1.4 | 1.1 |
| DBN-PhPym | 523 | 34 | 99.0 | 92.8 | 6.2 | 8.6 | 44.0 | 10.8 | 0.1 | 0.7 | 2.2 | 2.2 |

^a)^ PL emission maximum. ^b)^ Full width at half maximum of the PL spectrum. ^c)^ The total photoluminescence quantum yield (*Φ*_PL_). ^d)^ The prompt fluorescent (*Φ*_F_) component of *Φ*_PL_. ^e)^ The delayed fluorescent (*Φ*_TADF_) component of *Φ*_PL_. ^f)^ The lifetime of prompt fluorescence (*τ*_F_). ^g)^ The lifetime of delayed fluorescence (*τ*_TADF_). ^h)^ The rate constant of prompt fluorescence (*k*_F_). ^i)^ The rate constant of internal conversion (*k*_IC_). ^j)^ The rate constant of intersystem crossing (*k*_ISC_). ^k)^ The rate constant of TADF (*k*_TADF_). ^l)^ The rate constant of reverse intersystem crossing (*k*_RISC_).

**Table S2.** Summary of the EL data of the non-sensitized devices based on DBN-Pym and DBN-PhPym.

| Emitter | *x wt%* | *λ*_em_  [nm]^a)^ | FWHM  [nm]^a)^ | L_max_  [cd m^-2^] | V_on_ [V]^b)^ | CE_max_  [cd A^-1^] | PE_max_  [lm W^-1^] | EQE  [%]^c)^ | CIE  [x,y]^a)^ |
| --- | --- | --- | --- | --- | --- | --- | --- | --- | --- |
| DBN-Pym | 2 | 527 | 32 | 13955 | 3.3 | 41.6 | 39.6 | 10.3/ 6.6/ 3.9 | (0.275, 0.687) |
|  | 5 | 529 | 40 | 10512 | 3.3 | 32.2 | 30.7 | 8.0/ 4.8/ 2.8 | (0.307, 0.660) |
|  | 10 | 531 | 41 | 5910 | 3.3 | 14.9 | 14.2 | 3.8/ 2.6/ 1.7 | (0.311, 0.656) |
| DBN-PhPym | 2 | 523 | 33 | 16633 | 2.7 | 46.9 | 54.6 | 12.1/ 7.5/ 5.7 | (0.224, 0.706) |
|  | 5 | 526 | 33 | 14792 | 2.7 | 40.7 | 47.4 | 10.2/ 6.2/ 4.4 | (0.250, 0.700) |
|  | 10 | 527 | 34 | 7907 | 2.7 | 31.5 | 36.6 | 7.8/ 4.2/ 2.8 | (0.276, 0.685) |

^a)^ EL spectra measured at 1000 cd m^-2^. ^b)^ Turn-on voltage at around 1 cd m^-2^. ^c)^ Maximum external quantum efficiency, and values at 1000 and 10000 cd m^-2^, respectively.

**Table S3.** Summary of the EL data of the sensitized device based on SBN-PhPym.

| Emitter | *x wt%* | *λ*_em_  [nm]^a)^ | FWHM  [nm]^a)^ | L_max_  [cd m^-2^] | V_on_ [V]^b)^ | CE_max_  [cd A^-1^] | PE_max_  [lm W^-1^] | EQE  [%]^c)^ | CIE  [x,y]^a)^ |
| --- | --- | --- | --- | --- | --- | --- | --- | --- | --- |
| SBN-PhPym | 2 | 519 | 41 | 18433 | 3.3 | 94.9 | 58.6 | 25.1/ 24.6/ 18.4 | (0.246, 0.672) |

^a)^ EL spectra measured at 1000 cd m^-2^. ^b)^ Turn-on voltage at around 1 cd m^-2^. ^c)^ Maximum external quantum efficiency, and values at 1000 and 10000 cd m^-2^, respectively.

**Table S4.** Summary of the EL data of the non-sensitized devices (using mCP host matrix) based on DBN-Pym and DBN-PhPym.

| Emitter | *x wt%* | *λ*_em_  [nm]^a)^ | FWHM  [nm]^a)^ | L_max_  [cd m^-2^] | V_on_ [V]^b)^ | CE_max_  [cd A^-1^] | PE_max_  [lm W^-1^] | EQE  [%]^c)^ | CIE  [x,y]^a)^ |
| --- | --- | --- | --- | --- | --- | --- | --- | --- | --- |
| DBN-Pym | 2 | 528 | 34 | 726 | 4.2 | 32.1 | 22.4 | 7.9 | (0.283, 0.681) |
| DBN-PhPym | 2 | 524 | 34 | 758 | 3.6 | 36.7 | 27.8 | 8.8 | (0.240, 0.691) |

^a)^ EL spectra measured at 100 cd m^-2^. ^b)^ Turn-on voltage at around 1 cd m^-2^.

**Table S5.** Reported Solution-Processed narrowband MR- TADF emitters *vs* this work.

|  | Emitter | *λ*_EL_  [nm] | EQE_max_  [%] | EQE_1000 cd/m2_  [%] | FWHM  [nm] | CIE  (x,y) | Ref. |
| --- | --- | --- | --- | --- | --- | --- | --- |
|  | **DBN-Pym** | **526** | **19.9** | **19.8** | **32** | **(0.256,0.690)** | **This work** |
|  | **DBN-PhPym** | **521** | **29** | **28.3** | **34** | **(0.213,0.693)** | **This work** |
| 1 | Endo-D2 | 472 | 22.6 | - | 38 | (0.13,0.22) | *Angew. Chem. Int. Ed.* **2024**, *64*, e202415607 |
| 2 | *v*-DABNA | 472 | 16.6 | 14.4 | 23 | (0.14, 0.18) | *J. Mater. Chem. C,* **2022**,*10,* 4590 |
| 3 | 2TCzBN | 480 | 23.1 | 22.2 | 36 | (0.14, 0.34) | *Adv. Funct. Mater.* **2023**, *33,* 2301327 |
| 4 | *v*-DABNA-Mes | 480 | 22.9 | 10.9 | 27 | (0.09, 0.21) | *J. Am. Chem. Soc.* **2022***, 144,* 106−112 |
| 5 | Cz-DABNA | 472 | 23.8 | 21.2 | 17 | (0.138, 0.190) | *Angew. Chem. Int. Ed.* **2023**, *62*, e202313084 |
| 6 | t-BuCz-DABNA | 472 | 29.2 | 26 | 17 | (0.139, 0.189) |  |
| 7 | DtBuCzB | 486 | 23.9 | 21.5 | 28 | (0.12, 0.38) | *Adv. Optical Mater.* **2023***, 11,* 2201898 |
| 8 | TriBNCz | 496 | 28.8 | 26.1 | 30 | (0.097, 0.434) | *Chem. Eng. J.* **2024**, *493, 152857* |
| 9 | BCzBN | 490 | 16.3 | - | 32 | - | *Chin. Chem. Lett.* **2021**, *32*, 1372–1376 |
| 10 | CzBN | 480 | 14.7 | - | 35 | - |  |
| 11 | BN-36Cz-BN | 495 | 27.1 | 2.9 | 31 | (0.11, 0.50) | *Angew. Chem. Int. Ed.* **2023**, *62*, e202301988 |
| 12 | BN-27Cz-BN | 498 | 20.9 | 3.3 | 33 | (0.14,0.58) |  |
| 13 | 6TBN | 496 | 23 | 6.4 | 25 | (0.09, 0.52) | *ACS Appl. Mater. Interfaces* **2023**, *15*, 39669-39676 |
| 14 | BN-CP1 | 492 | 26.6 | 3.6 | 28 | (0.14, 0.46) | *Adv. Mater.* **2023***, 35,* 2300510 |
| 15 | TCzBN-S | 500 | 23.3 | 6.7 | 39 | (0.13, 0.59) | *Chem. Eng. J.* **2023**, *465*, 142900 |
| 16 | TCzBN-SO | 516 | 25.5 | 6.9 | 41 | (0.19, 0.68) |  |
| 17 | OAB-ABP-1 | 505 | 21.8 | 17.4 | 33 | (0.12, 0.63) | *Adv. Mater.* **2020**, *32*, 2004072 |
| 18 | BNCPPt | 507 | 13.5 | 12.9 | 35 | (0.22, 0.63) | *Chem. Eng. J.* **2023**, *478*, 147123 |
| 19 | BN4 | 512 | 19 | 10.1 | 49 | (0.206, 0.635) | *Adv. Mater.* **2022***, 34,* 2105080 |
| 20 | BN5 | 506 | 26.5 | 11.1 | 48 | (0.167, 0.603) |  |
| 21 | *m*-Cz-BNCz | 519 | 24.2 | 17.7 | 42 | (0.20, 0.67) | *Chem. Eng. J.* **2022**, *447*, 137517 |
| 22 | 3CzSF-BN | 519 | 23.1 | 21.3 | 37 | (0.201,0.710) | *Adv. Optical Mater.* ***2024****, 12, 2400490* |
| 23 | BDP-2 | 522 | 21.5 | - | 31 | (0.28,0.65) | *Mater. Horiz.* **2024**, *11*, 6126-6140 |
| 24 | BF-G | 524 | 30.7 | 28.5 | 36 | (0.27,0.67) | *Angew. Chem. Int. Ed.* ***2024****,* 63, *e202412720* |
| 25 | QAO-DAd | 556 | 19.3 | - | - | (0.41,0.56) | *Adv. Optical Mater.* **2019***, 7,* 1801536 |
| 26 | BN-R1 | 611 | 19 | 9.1 | 44 | (0.640, 0.359) | *Adv. Optical Mater.* **2023**, *12*, 2302811 |
| 27 | BN-R2 | 601 | 20.4 | 9.9 | 41 | (0.606, 0.393) |  |
| 28 | BN-R3 | 587 | 20.4 | 17.5 | 44 | (0.553, 0.444) |  |
| 29 | BN-R | 618 | 22 | 13.3 | 46 | (0.65, 0.34) | *Angew. Chem. Int. Ed.* **2023**, *62*, e202216473 |

1. **References**

[S1] A. D. Becke, *J. Chem. Phys.* **1993**, *98*, 5648.

[S2] C. Lee, W. Yang, R. G. Parr, *Phys. Rev. B* **1988**, *37*, 785.

[S3] R. Krishnan, J. S. Binkley, R. Seeger, J. Pople, *J. Chem. Phys.* **1980**, *72*, 650.

[S4] S. Grimme, J. Antony, S. Ehrlich, H. Krieg, *J. Chem. Phys.* **2010**, *132*, 154104.

[S5] Gaussian 09, Revision D.01, M. J. Frisch, G. W. Trucks, H. B. Schlegel, G. E. Scuseria, M. A. Robb, J. R. Cheeseman, G. Scalmani, V. Barone, B. Mennucci, G. A. Petersson, H. Nakatsuji, M. Caricato, X. Li, H. P. Hratchian, A. F. Izmaylov, J. Bloino, G. Zheng, J. L. Sonnenberg, M. Hada, M. Ehara, K. Toyota, R. Fukuda, J. Hasegawa, M. Ishida, T. Nakajima, Y. Honda, O. Kitao, H. Nakai, T. Vreven, J. A. Montgomery, Jr., J. E. Peralta, F. Ogliaro, M. Bearpark, J. J. Heyd, E. Brothers, K. N. Kudin, V. N. Staroverov, T. Keith, R. Kobayashi, J. Normand, K. Raghavachari, A. Rendell, J. C. Burant, S. S. Iyengar, J. Tomasi, M. Cossi, N. Rega, J. M. Millam, M. Klene, J. E. Knox, J. B. Cross, V. Bakken, C. Adamo, J. Jaramillo, R. Gomperts, R. E. Stratmann, O. Yazyev, A. J. Austin, R. Cammi, C. Pomelli, J. W. Ochterski, R. L. Martin, K. Morokuma, V. Zakrzewski, G. A. Voth, P. Salvador, J. J. Dannenberg, S. Dapprich, A. D. Daniels, O. Farkas, J. B.

Foresman, J. V. Ortiz, J. Cioslowski, and D. J. Fox, Gaussian, Inc., Wallingford CT, **2013**.

[S6] T. Lu, F. Chen, *J. Comput. Chem.*, **2012**, *33*, 580-592.

[S7] Q. Zhang, H. Kuwabara, W. J. Potscavage, S. Huang, Y. Hatae, T. Shibata, C. Adachi, *J. Am. Chem. Soc.* **2014**, *136*, 18070.

[S8] Q. Zhang, B. Li, S. Huang, H. Nomura, H. Tanaka, C. Adachi, *Nat. Photonics* **2014**, *8*, 326.

[S9] T.-L. Wu, M.-J. Huang, C.-C. Lin, P.-Y. Huang, T.-Y. Chou, R.-W. Chen-Cheng, H.-W. Lin, R.-S. Liu, C.-H. Cheng, *Nat. Photonics* **2018**, *12*, 235.
